# Accurate Measurement of the Effects of All Amino-Acid Mutations on Influenza Hemagglutinin

**DOI:** 10.3390/v8060155

**Published:** 2016-06-03

**Authors:** Michael B. Doud, Jesse D. Bloom

**Affiliations:** 1 Division of Basic Sciences Basic Sciences and Computational Biology Program, Fred Hutchinson Cancer Research Center, 1100 Fairview Ave N, Seattle, WA 98109, USA; mbdoud@gmail.com; 2 Department of Genome Sciences, University of Washington, 3720 15th Ave NE, Seattle, WA 98195-5065, USA; 3 Medical Scientist Training Program, University of Washington, Seattle, WA 98109, USA

**Keywords:** influenza, hemagglutinin, mutational tolerance, deep mutational scanning, evolution

## Abstract

Influenza genes evolve mostly via point mutations, and so knowing the effect of every amino-acid mutation provides information about evolutionary paths available to the virus. We and others have combined high-throughput mutagenesis with deep sequencing to estimate the effects of large numbers of mutations to influenza genes. However, these measurements have suffered from substantial experimental noise due to a variety of technical problems, the most prominent of which is bottlenecking during the generation of mutant viruses from plasmids. Here we describe advances that ameliorate these problems, enabling us to measure with greatly improved accuracy and reproducibility the effects of all amino-acid mutations to an H1 influenza hemagglutinin on viral replication in cell culture. The largest improvements come from using a helper virus to reduce bottlenecks when generating viruses from plasmids. Our measurements confirm at much higher resolution the results of previous studies suggesting that antigenic sites on the globular head of hemagglutinin are highly tolerant of mutations. We also show that other regions of hemagglutinin—including the stalk epitopes targeted by broadly neutralizing antibodies—have a much lower inherent capacity to tolerate point mutations. The ability to accurately measure the effects of all influenza mutations should enhance efforts to understand and predict viral evolution.

## 1. Introduction

Seasonal influenza is a recurrent threat to human health, largely because it rapidly accumulates amino-acid mutations in proteins targeted by the immune system [1]. Measuring the functional impact of every possible amino-acid mutation to influenza can therefore provide useful information about which evolutionary paths are accessible to the virus. Such measurements are now possible using deep mutational scanning [2,3]. When applied to influenza, this technique involves creating all codon mutants of a viral gene, incorporating these mutant genes into viruses that are subjected to a functional selection, and estimating the functional impact of each mutation by using deep sequencing to quantify its frequency pre- and post-selection. We and others have used deep mutational scanning to estimate the effects of all amino-acid [4,5,6] or nucleotide [7,8] mutations to several influenza genes, and Heaton and coworkers [9] have used a similar approach to examine influenza’s tolerance to short insertions. However, these studies suffered from substantial noise that degrades the utility of their results. For instance, in every study that reported the results for independent experimental replicates, the replicate-to-replicate correlation was mediocre.

This experimental noise arises primarily from bottlenecking of mutant diversity during the generation of viruses from plasmids. The influenza genome consists of eight negative-sense RNA segments. During viral infection, gene expression from these segments is a highly regulated process [10,11,12]. Generating influenza from plasmids involves co-transfecting mammalian cells with multiple plasmids that must yield all eight viral gene segments and at least four viral proteins at a stoichiometry that leads to assembly of infectious virions [13,14,15]. This plasmid-driven process is understandably less efficient than viral infection. A small fraction of transfected cells probably yield most initial viruses, which are then amplified by secondary infection. This bottlenecking severely hampers experiments that require creating a diverse library of viruses from an initial library of plasmids.

Several strategies have been used to overcome problems associated with bottlenecks during the generation of influenza from plasmids. One strategy is to generate and titer each viral variant individually, and then mix them [16,17]. A second strategy is to reduce the impact of bottlenecks by shrinking the complexity of the libraries, such as by only mutating a small portion of a viral gene [18,19]. Neither of these strategies scale effectively to the deep mutational scanning of full-length proteins, since there are ∼10${}^{4}$ unique amino-acid mutants of a 500-residue protein.

To overcome these limitations, we have developed a novel approach that uses a “helper virus” to generate virus libraries without strong bottlenecking. We have combined this approach with other technical improvements to perform deep mutational scanning of all amino-acid mutations to an H1 hemagglutinin (HA) with much higher accuracy and reproducibility than existing deep mutational scans of influenza genes. We use phylogenetic analyses to show that our measurements accurately reflect constraints on HA evolution in nature. We confirm that antigenic sites in the globular head of HA are highly tolerant of mutations, and identify other regions of the protein that are more constrained. These advances improve our understanding of HA’s inherent evolutionary capacity and can help inform evolutionary modeling and guide the development of vaccines targeting sites with a limited capacity for mutational escape.

## 2. Results

### 2.1. A Helper-Virus Enables Efficient Production of Mutant Virus Libraries from Plasmids

**Figure 1:**
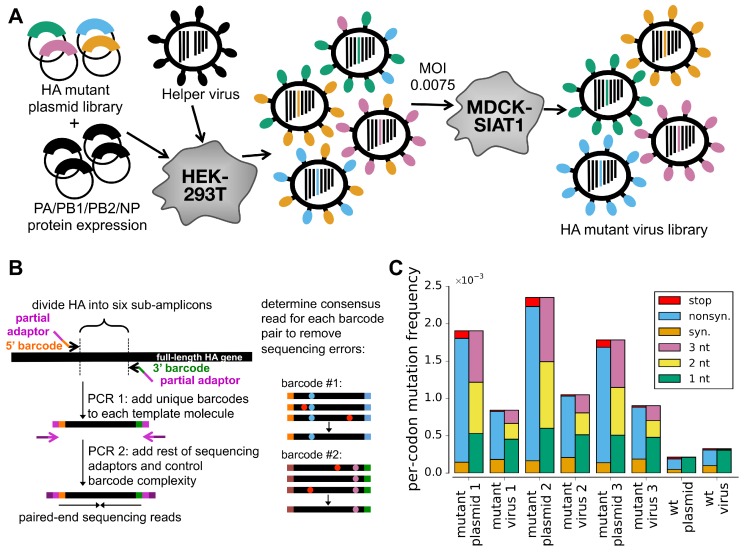
**Deep mutational scanning of HA**. (**A**) Cells transfected with a plasmid mutant library of HA are infected with an HA-deficient helper virus to yield a library of mutant viruses. This virus library is passaged at low MOI to select for functional variants and enforce genotype-phenotype linkage. The helper viruses themselves are propagated in cells constitutively expressing HA (Figure S1). The variants in the plasmid mutant library contain an average of one codon mutation, with the number of mutations per clone following a roughly Poisson distribution (Figure S2). The helper-virus works best when HA is provided on a plasmid that directs the synthesis of only viral RNA (Figure S3). (**B**) Accurate Illumina sequencing using barcoded subamplicons. HA is divided into six sub-amplicons, and a first round of PCR appends random barcodes and part of the Illumina adaptor to each subamplicon. The complexity of these barcoded subamplicons is controlled to be less than the sequencing depth, and a second round of PCR adds the remaining adaptor. Sequencing reads are grouped by barcode, distinguishing sequencing errors that occur in only one read (red dots) from true mutations that occur in all reads (blue and purple dots). (**C**) The overall mutation frequencies reveal selection against stop codons and many nonsynonymous mutations in the mutant viruses relative to the plasmids from which they were generated (see also Figure S4). Sequencing of unmutated plasmid and virus generated from this plasmid (denoted as “wt plasmid” and “wt virus” in panel C) indicates rates of sequencing, reverse-transcription, and viral replication errors are lower than the mutation rates in the libraries, enabling us to reliably distinguish the signal and noise.

We reasoned that the process of generating viral libraries carrying HA mutants would be more efficient if transfected cells only needed to produce HA from plasmid, and the other gene segments and proteins were delivered by viral infection (Figure 1A). The Palese lab has previously shown that a seven-segmented HA-deficient virus can be propagated in cells that constitutively express HA protein [20]. We created HA-expressing cells and validated that we could propagate an HA-deficient A/WSN/1933 (H1N1) virus (Figure S1).

We cloned triplicate plasmid libraries of random codon mutants of the A/WSN/1933 HA gene. These libraries contain multi-nucleotide (e.g., GGC→CAT) as well as single-nucleotide (e.g., GGC→GAC) codon mutations. There are $63\times 565\approx 3.5\times 10^{4}$ different codon mutations that can be made to the 565-codon HA gene, corresponding to $19\times 565\approx 10^{4}$ amino-acid mutations. The deep sequencing described below found at least three occurrences of over 97% of these amino-acid mutations in each of the three replicate plasmid mutant libraries. These libraries have a somewhat lower mutation rate than our previous deep mutational scan of hemagglutinin [4], with the number of mutations per clone following a roughly Poisson distribution with a mean of about one (Figure S2). We cloned these HA libraries into both uni-directional and bi-directional reverse-genetics plasmids [13,14].

We then transfected cells with one of the HA plasmid mutant libraries along with plasmids expressing the four viral polymerase-related proteins (PB2, PB1, PA, and NP) with the goal of generating pre-formed viral ribonucleoprotein complexes carrying the HA segment. These transfected cells were then infected with the HA-deficient helper virus, and 24 h later, we determined the titer of fully competent virus in the supernatant. The highest titers (∼10${}^{3}$ TCID${}_{50}$ per μL) were obtained using the uni-directional reverse-genetics plasmid (Figure S3). The reason that we co-transfected protein expression plasmids for the four polymerase-related proteins was to create pre-formed viral ribonucleoprotein complexes. Virus titers were ∼100-fold lower when the polymerase plasmids were not co-transfected (data not shown). Overall, these findings demonstrate the feasibility of the helper-virus strategy in Figure 1A.

We next used this helper-virus strategy to independently generate three mutant virus libraries, one from each of our triplicate plasmid mutant libraries. Each mutant virus library should sample most of the codon mutations to the A/WSN/1933 HA. We also generated a control virus library from a plasmid encoding the unmutated wild-type HA gene.

### 2.2. Low MOI Passage Combined with Barcoded-Subamplicon Sequencing Reveals Strong Selection against Non-Functional HA Variants

To select for viruses carrying functional HA variants, we passaged the mutant virus libraries at a low multiplicity of infection (MOI) of 0.0075 TCID${}_{50}$ per cell as outlined in Figure 1A. This MOI is substantially lower than the MOI of 0.1 that we used in our previous study to examine the effects of all mutations to HA [4], and was chosen with the goal of more effectively purging non-functional HA variants.

To quantify selection on HA, we needed our deep sequencing to be sufficiently accurate to determine the frequency of each mutation pre- and post-selection. Standard Illumina sequencing has an error rate that is too high. In our previous deep mutational scanning of influenza [4,5,6], we reduced this error rate by using overlapping paired-end reads. Here, we used an alternative error-correction strategy that involves attaching random barcodes to PCR subamplicons and then clustering reads with the same barcode (Figure 1B). To our knowledge, this basic strategy was first described by Hiatt *et al.* [21] and first applied to influenza by Wu *et al.* [7]. Sequencing of the unmutated plasmid allows us to estimate that the error rate is ∼2 × 10${}^{-4}$ per codon, corresponding to <10${}^{-4}$ per nucleotide (Figure 1C, sample referred to as “wt plasmid”). This error rate is substantially lower than we obtained previously using overlapping paired-end reads, consistent with the results of the sequencing-strategy comparison by Zhang *et al.* [22]. Sequencing of viruses generated from the unmutated plasmid shows that the error rates associated with reverse-transcription and viral replication are also tolerably low (below the mutation rate in the mutant libraries) (Figure 1C, sample referred to as “wt virus”).

Figure 1C reveals strong selection against non-functional HA variants. The plasmid mutant libraries contain a mix of synonymous, nonsynonymous, and stop-codon mutations. However, stop-codon mutations are almost completely purged from the passaged mutant virus libraries, as are many nonsynonymous mutations. The selection against the stop codons is stronger than in our previous deep mutational scan [4] (Figure S4). Overall, these results indicate strong selection on HA that can be quantified by accurate deep sequencing.

### 2.3. The Mutant Virus Libraries Have Reduced Bottlenecking and Yield Reproducible Measurements of Mutational Effects

**Figure 2:**
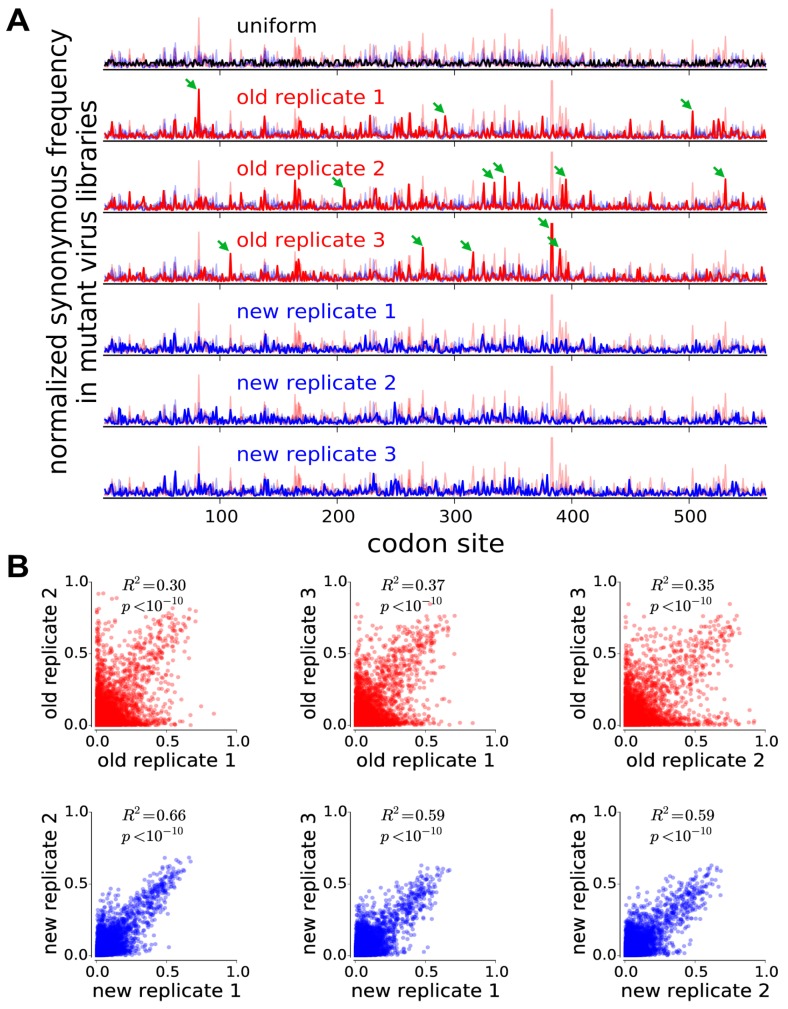
**The use of helper viruses increases reproducibility due to reduced bottlenecking during the generation of the mutant virus libraries.** (**A**) Each row shows the synonymous mutation frequency for every site normalized to the total synonymous frequency for that sample. If synonymous mutations are sampled uniformly, the data should resemble the black line in the top row (the line is not completely straight because different codons have different numbers of synonymous variants). The next six rows show the synonymous mutation frequencies for each replicate of the old (**red lines**) [4] and new (**blue lines**) experiments. To assist in comparing the locations and heights of peaks across all samples, the data for each replicate are shown as a thick line in front of thin lines representing the other five replicates. The old experiments have more bottlenecking as manifested by taller peaks indicating synonymous mutations that were stochastically enriched in each replicate (examples marked by **green arrows**). The differences between replicates are *not* due to differences in synonymous mutation frequencies in the plasmid libraries used to generate the viruses (Figure S5). (**B**) The mutational effects measured in the new experiments are much more reproducible across replicates. Each plot shows the squared Pearson correlation coefficient for all site-specific amino-acid preferences measured in a pair of independent experimental replicates.

To evaluate whether the virus libraries were bottlenecked, we examined the distribution of synonymous mutation frequencies in each library. If bottlenecking causes a few mutants to stochastically dominate, we expect that in each library a few sites will have relatively high synonymous mutation frequencies and that these sites will differ among replicates. Figure 2A shows normalized synonymous mutation frequencies across HA for each of the three replicate mutant virus libraries from both our previous deep mutational scan of HA that utilized reverse genetics [4], and the current study utilizing helper viruses. In the older study, each replicate had a different handful of sites with greatly elevated synonymous frequencies (green arrows), indicative of stochastic bottlenecking. In contrast, in our new virus libraries, the distribution of synonymous mutation frequencies is much more uniform across the HA gene. Specifically, the standard deviation of normalized synonymous frequencies was 1.63 ± 0.14 for the old libraries, but only 1.18 ± 0.05 for the new libraries, indicating less bottlenecking-induced variation in mutation frequencies in the new libraries.

We next evaluated the reproducibility of our measurements of the effects of each amino-acid mutation. We estimated the effect of each mutation from its change in frequency in the mutant viruses relative to the original plasmid libraries, correcting for the site-specific error rates determined by sequencing unmutated virus and plasmid, and performing the analyses using the algorithms described in [23] and implemented in the dms_tools software (version 1.1.12, available at http://jbloomlab.github.io/dms_tools/). The results are quantified in terms of the *preference* of each site for each amino-acid; the set of all 20 preferences at a site can be thought of as representing the expected post-selection frequency of each amino acid at that site if all amino acids are initially present at equal frequencies.

Figure 2B shows the correlation between the amino-acid preferences from each experimental replicate. The replicate-to-replicate reproducibility is dramatically improved in our new experiments relative to our previous work utilizing reverse genetics [4], with the average Pearson’s $R^{2}$ increasing from 0.34 to 0.61. The new experiments are also largely free of the most problematic type of noise that plagued the previous study, where an amino acid at a site is deemed highly preferred in one replicate but disfavored in another. Overall, these results demonstrate that our new strategies enable more reproducible measurement of the effects of all mutations to HA.

### 2.4. The Measurements Better Reflect the Constraints on HA Evolution in Nature

We next tested whether our new measurements better describe the evolution of HA in nature. The accuracy with which experimental measurements of site-specific amino-acid preferences reflect the constraints shaping a protein’s evolution in nature can be quantified by comparing the phylogenetic fit of experimentally informed substitution models [5]. We assembled a set of human and swine influenza HA sequences and fit substitution models using phydms [24] (version 1.1.1, available at http://jbloomlab.github.io/phydms/), which in turn uses Bio++ [25] for the likelihood calculations.

A substitution model informed by our new measurements described the natural evolution of HA better than a model informed by our older measurements from [4], and vastly better than conventional non-site-specific substitution models (Table 1). Averaging the measurements from both studies improved phylogenetic fit even further, a finding consistent with previous work reporting that combining data from multiple deep mutational scanning studies of the same protein tends to improve substitution models [6].

**Figure 3:**
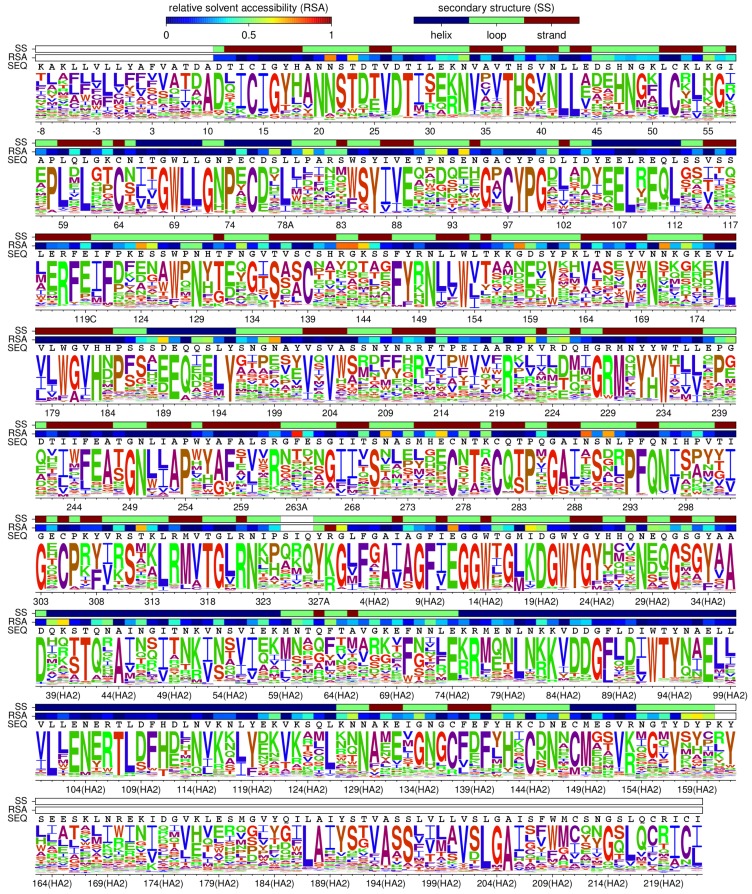
**HA’s site-specific amino-acid preferences.** The preference of each site in HA for each of the 20 amino-acids as inferred by combining the new and old data and re-scaling by the stringency parameter inferred in Table 1. The height of each letter is proportional to the preference for that amino acid at that site. The overlay bars show each residue’s secondary structure, relative solvent accessibility, and wildtype identity in the A/WSN/1933 HA. Amino acids are colored according to hydrophobicity using a blue-to-red-to-green scale (most hydrophobic is **blue**, most hydrophilic is **green**). The sequence is numbered using the H3 numbering scheme. Conversion between WSN sequential numbering and H3 numbering is provided in File S6.

The phylogenetic model fitting optimizes a parameter that accounts for differences in the stringency of selection between the experiments and natural evolution [29]; a stringency parameter >1 indicates that natural selection prefers the same amino acids as the experimental selections but with greater strength. The best model in Table 1 has a stringency parameter of 1.8. The site-specific amino-acid preferences for this model scaled by this stringency parameter are displayed in Figure 3; text files with unscaled and scaled numerical values are in File S2 and File S3.

### 2.5. A Handful of Sites Are under Very Different Selection in Our Experiments Than in Nature

**Figure 4:**
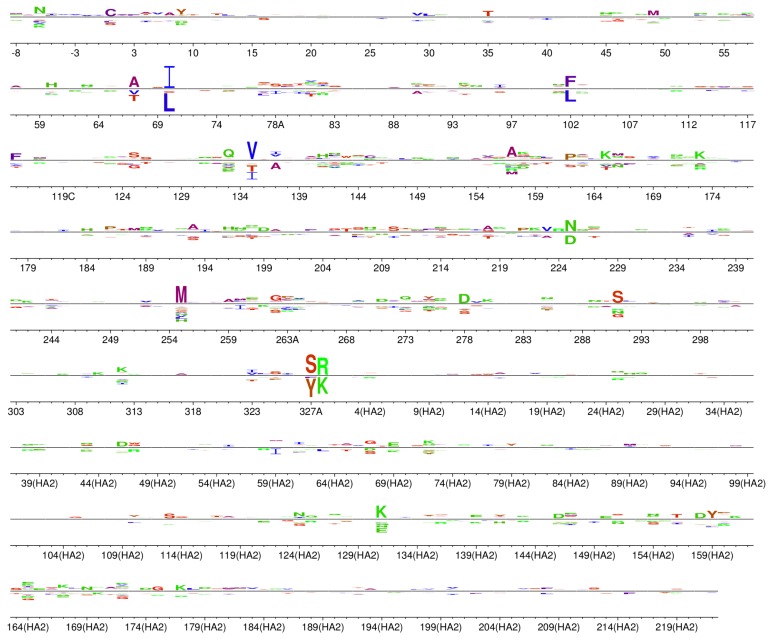
**Differential selection between our selection in the lab and HA’s evolution in nature.** The approach in [24] was used to determine the magnitude of differential selection between the deep mutational scanning and the natural evolution of human seasonal H1N1 and classical swine H1N1 HAs. At each site, the height of a letter above or below the center line indicates that differential selection for or against that amino acid in nature as compared to our experiments. At most sites, the differential selection is very small, showing that the experimental measurements are mostly concordant with natural selection on HA. However, a few sites are under very different selection in nature as compared to our experiments. The sequence is numbered using the H3 numbering scheme. Amino acids are colored by hydrophobicity as in Figure 3. Conversion between WSN sequential numbering and H3 numbering is provided in File S6.

We next asked whether there are sites in HA that evolve in nature in a way that is highly discordant with our experimental measurements. To do this, we again used phydms [24] to identify selection in nature for amino acids that differ from the ones preferred in the deep mutational scanning, again using natural sequences from seasonal human H1N1 and classical swine H1N1 HAs. Briefly, this program uses a maximum-likelihood phylogenetics approach to estimate the difference in preference for each amino acid at each site between the experimental measurements and selection in nature (see [24] for details). Figure 4 shows the difference in amino-acid preferences between our experiments and natural evolution for each site in HA. At most sites, the magnitude of differential selection is small, indicating that the experimentally measured preferences mostly parallel constraints on natural evolution. Sites that are under strong differential selection usually show conservative changes; for example, site 70 (H3 numbering) prefers isoleucine in nature but leucine in our deep mutational scanning.

One of the most striking exceptions to this general concordance between natural selection and our experiments can be given a clear explanation. At site 327A (H3 numbering), the experimentally measured preference for tyrosine is at odds with nature’s strong preference for serine (Figure 4). The lab-adapted A/WSN/1933 strain used in our experiments differs from naturally occurring influenza in that it uses plasmin to cleave and activate HA [30,31]. Plasmin cleavage is enhanced by tyrosine at this site [32], so it is unsurprising that our experiments detected a preference at this site unique to the influenza strain we used. This example illustrates how the occasional deviations from the general concordance between deep mutational scanning experiments and natural selection can point to interesting biological mechanisms.

### 2.6. Antigenic Sites in HA’s Globular Head Are Highly Tolerant of Mutations, but Stalk Epitopes Targeted by Broadly Neutralizing Antibodies Are Not

**Figure 5:**
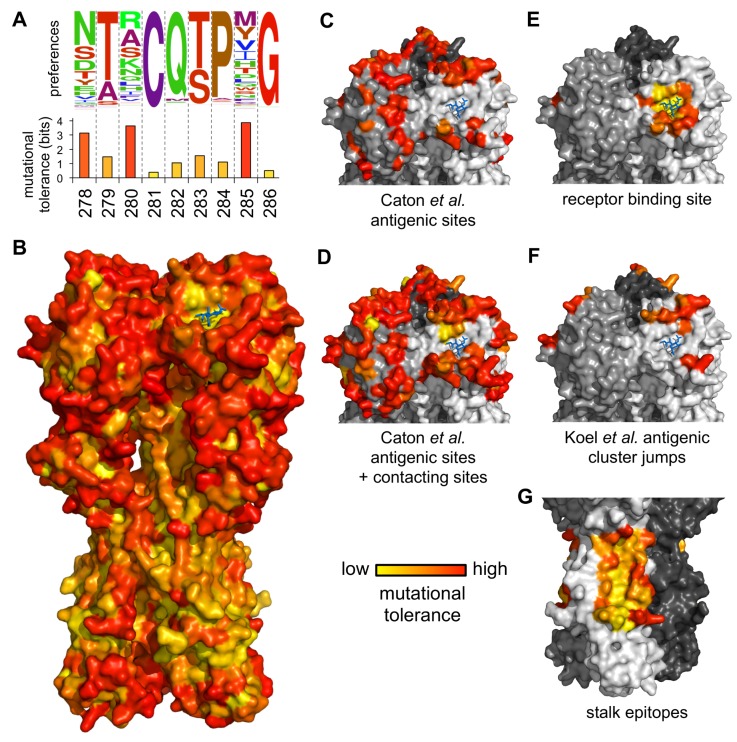
**Antigenic sites in HA’s globular head have a high inherent tolerance for mutations, but HA’s stalk is relatively intolerant of mutations.** (**A**) Mutational tolerance is calculated as the Shannon entropy of a site’s amino-acid preferences. (**B**) Mutational tolerance mapped onto the HA trimer (**yellow** indicates low tolerance, **red** indicates high tolerance, **blue** sticks show the sialic-acid receptor). (**C**,**D**) The antigenic sites defined by Caton *et al.* [33] have high mutational tolerance, as do the residues contacting these sites. (**E**) Conserved receptor-binding residues have low mutational tolerance. (**F**) Sites that contribute to antigenic cluster jumps [34]. (**G**) Sites in the footprints of four broadly neutralizing antibodies have low mutational tolerance. Shown are footprints of F10, CR6261, FI6v3, and CR9114 [35,36,37,38]. For panels B-G, tolerance is mapped onto PDB structure 1RVX [39]. For panels C-G, each monomer is shown in a different shade of gray. Figure S6 reports statistical analyses of whether subsets of sites have higher or lower tolerance than expected given their solvent accessibility. Figure S7 shows the tolerance of the different domains of HA.

We computed the inherent mutational tolerance of each site using the stringency-scaled amino-acid preferences from the combined datasets (Figure 5A). The mutational tolerance is mapped onto the structure of HA in Figure 5B.

The H1 HA antigenic sites defined by Caton *et al.* [33] are significantly more mutationally tolerant than the average site (Figure 5C), even after accounting for relative solvent accessibility (Figure S6A). This high mutational tolerance extends to other solvent-exposed residues in contact with the antigenic sites (Figure 5D, Figure S6B), indicating that the HA molecular surfaces commonly targeted by antibodies have a high inherent capacity for evolutionary change. This high mutational tolerance does not extend to the receptor-binding pocket (Figure 5E, Figure S6C,D) but may be a feature of the sites that make the greatest contributions to the punctuated antigenic evolution of H3N2 and seasonal H1N1 HA [34] (Figure 5F), albeit not at a level that is statistically significant after correcting for solvent accessibility (Figure S6E). These results support the findings of our previous study [4] that the sites in HA that are the immunodominant targets of antibodies have a high inherent capacity to tolerate mutations.

Perhaps in part because of the high mutational tolerance of the antigenic sites in its globular head, HA is adept at escaping antibody-mediated immunity [1,40]. New vaccines are being developed that aim to elicit immunity against other portions of HA [41], most commonly regions in the stalk that are relatively conserved among naturally occurring strains. An important question is whether these stalk regions are conserved because they are inherently intolerant of point mutations, or simply because they are not currently under immune pressure. To answer this question, we examined the inherent mutational tolerance of the largely overlapping epitopes of four broadly neutralizing anti-stalk antibodies: F10 [35], CR6261 [36], FI6v3 [37], and CR9114 [38]. Visual inspection of Figure 5G shows that these stalk epitopes have a low mutational tolerance, a result that is confirmed by statistical analysis (Figure S6F). Therefore, the epitopes that next-generation vaccines aim to target indeed have a reduced capacity for immune escape by point mutations. This finding is also consistent with Heaton *et al.*’s report that HA’s stalk is intolerant to insertions [9].

We wondered if some of HA’s variation in mutational tolerance is explained by differences in the three ancient domains that compose the protein. HA is the product of a series of ancient insertions that merged a fusion domain, a receptor-binding domain (which contains the majority of the antigenic sites as well as the receptor-binding pocket itself), and a vestigial esterase domain [42]. We compared the inherent mutational tolerance of these three domains, again correcting for solvent accessibility. We found that sites in the receptor-binding domain on average have a significantly higher mutational tolerance than all sites in the protein, although sites in the receptor-binding pocket itself are often highly constrained (Figure 5, Figure S7). On the other hand, sites in the fusion domain have a significantly lower mutational tolerance than all sites (Figure S7). This enriched tolerance to point mutations throughout the receptor-binding domain is also concordant with the results of Heaton *et al.*, showing that the receptor-binding domain is uniquely tolerant to short insertions [9]. Therefore, HA’s antigenic evolvability is not just a consequence of the immunodominant antigenic sites themselves having high mutational tolerance, but also because these sites are found within a protein domain that is intrinsically more mutable than the rest of HA.

## 3. Discussion

We have described new techniques that greatly improve the reproducibility of deep mutational scanning of influenza. The largest improvement appears to result from using a helper virus to generate virus mutant libraries without the bottlenecks that plague the creation of viruses purely from plasmids. We have used these techniques to more accurately measure the effects of all amino-acid mutations to HA. Our measurements confirm at greater precision and resolution the finding [4,9] that HA’s propensity for immune escape is underpinned by the high inherent mutational tolerance of the immunodominant receptor-binding domain. Our data also show that some regions of HA—including the stalk epitopes targeted by new broadly neutralizing antibodies— have a reduced capacity for evolutionary change.

In this study, we measured the effects of all mutations to the HA from a lab-adapted H1N1 strain. To what extent can these measurements be extrapolated to other HAs? Due to epistasis, the effects of mutations sometimes change as proteins evolve [43,44]. However, many aspects of mutational effects are often roughly conserved during evolutionary divergence: for instance, experiments have shown that the effects of mutations on stability are often quite similar among homologs, both for HA [45] and proteins more generally [46,47]. In a previous study, we used deep mutational scanning to estimate the effects of all mutations to two close homologs of influenza nucleoprotein, and found that only a few sites exhibited large qualitative changes in their amino-acid preferences [6]. Therefore, the limited existing experimental work on this topic suggests that site-specific amino-acid preferences will often be broadly similar among homologs of the same protein, but that there will also be some shifts that can have important implications for evolution. However, further systematic investigation of this question is needed to assess the extent that deep mutational scanning studies like the one reported here can be extrapolated across protein homologs.

Overall, our work demonstrates a method for making accurate large-scale measurements of the effects of mutations to influenza proteins. Our results offer insight into how protein-intrinsic mutational tolerance shapes influenza evolution, and provide a basis for using deep mutational scanning to improve quantitative models of viral evolution and understand virus-immune interactions.

## 4. Materials and Methods

### 4.1. Growth of HA-Deficient Helper Virus in HA-Expressing Cells

MDCK-SIAT1 cells (Sigma, 05071502) were engineered to constitutively express the HA protein of A/WSN/1933 (H1N1) under control of the EF1a promoter by lentiviral transduction. These newly created cells will be referred to as MDCK-SIAT1-EF1a-WSN-HA cells since they are MDCK-SIAT1 cells that we have engineered to express the WSN HA under an EF1a promoter. HA surface expression was validated by flow cytometry (Figure S1).

To generate HA-deficient helper viruses, we seeded co-cultures of 293T cells (obtained from the ATCC, number CRL-3216; seeded at $5\times 10^{5}$ cells per well) and MDCK-SIAT1-EF1a-WSN-HA cells ($5\times 10^{4}$ cells cells per well) in 6-well dishes in D10 media (DMEM supplemented with 10% heat-inactivated FBS, 2 mM L-glutamine, 100 U of penicillin/mL, and 100 μg of streptomycin/mL). After 24 h, we transfected these co-cultures with bidirectional reverse-genetics plasmids for the seven non-HA segments of the A/WSN/1933 virus (pHW181-PB2, pHW182-PB1, pHW183-PA, pHW185-NP, pHW186-NA, pHW187-M, and pHW188-NS) [13] plus a protein expression plasmid for WSN HA (pHAGE2-CMV-WSNHA, which importantly does *not* contain non-coding regions of the HA segment or a promoter for the transcription of negative-sense viral RNA). Transfection was performed with BioT transfection reagent (Bioland B01-02, Paramount, CA, USA) with each well receiving 250 ng of each plasmid. Twenty-two hours after transfection, we changed the media to WSN growth media (Opti-MEM supplemented with 0.5% heat-inactivated FBS, 0.3% BSA, 100 U of penicillin/mL, 100 μg of streptomycin/mL, and 100 μg of calcium chloride/mL). At 96 h post-transfection, we passed 400 μL of the transfection supernatant into 15-cm dishes containing $4\;\times \;10^{6}$ MDCK-SIAT1 cells (as a negative control) or MDCK-SIAT1-EF1a-WSN-HA cells in WSN growth media. HA-deficient helper virus could only be propagated in the HA-expressing cells as expected (Figure S1). We collected the expanded helper virus from these cells after 68 h, aliquoted, and froze aliquots at −80 ${}^{\circ }$C. We titered the helper virus in MDCK-SIAT1-EF1a-WSN-HA cells by TCID${}_{50}$. We obtained titers between $10^{3}$ and $10^{4}$ TCID${}_{50}$ per μL when titering in MDCK-SIAT1-EF1a-WSN-HA cells, and no cytopathic effect except with extremely concentrated helper virus in MDCK-SIAT1 cells (Figure S1).

### 4.2. HA Plasmid Mutant Libraries

Codon mutagenesis was performed as described in [4] except that we performed one overall round of the PCR mutagenesis to yield a lower mutation rate (Figure S2). Ligation and eletroporation were also performed as in [4], except that we cloned the inserts into both pHW2000 [13] and pHH21 [14] plasmid backbones. All steps were performed in triplicate. For each replicate, we pooled over 3 million transformants, cultured in LB for 3 h in shaking flasks at 37 ${}^{\circ }$C, and maxi-prepped plasmid libraries.

### 4.3. Generation of Mutant HA Virus Libraries from Mutant Plasmids and Helper Viruses

To generate mutant virus libraries, we transfected 293T cells with a DNA mixture containing one of the three pHH21-MutantHA libraries (or the wild-type pHH21-WSN-HA control) and protein expression plasmids for the four proteins that compose the ribonucleoprotein complex, using plasmids HDM-Nan95-PA, HDM-Nan95-PB1, HDM-Nan95-PB2, and HDM-Aichi68-NP [43]. Specifically, we plated 293T cells in D10 at a density of $8\times 10^{5}$ per well in 6-well plates, changed the media to fresh D10 after 16 h, and then four hours later transfected cells with 500 ng of the HA reverse-genetics plasmid plus 375 ng of each of the PA, PB1, PB2, and NP plasmids using BioT. Twenty-four hours after transfection, we infected the cells with HA-deficient helper virus by making an inoculum of $1.3\times 10^{3}$ TCID${}_{50}$ per μL in WSN growth media, aspirating the D10 media from the cells, and adding 2 mL of inoculum to each well. After 3 h, we removed the inoculum by aspiration and added 2 mL of WSN growth media supplemented with 5% D10. Twenty-four hours after helper virus infection, we collected the supernatants for each replicate, stored aliquots at −80 ${}^{\circ }$C, and titered in MDCK-SIAT1 cells. Of note, we found that helper viruses that had been passaged more than once in MDCK-SIAT1-EF1a-WSN-HA cells tended to become less effective at rescuing fully replication competent viruses following infection of transfected cells, so we exclusively used single-passage helper virus in these experiments.

We passaged these transfection supernatants to create a genotype-phenotype link and impose functional selection on HA. We passaged over $9\times 10^{5}$ TCID${}_{50}$ at an MOI of 0.0075 TCID${}_{50}$ per cell. Specifically, for each library, we plated ten 15-cm dishes with $6\times 10^{6}$ MDCK-SIAT1 cells per dish and allowed cells to grow for 20 h, at which point they had reached a density $∼1.25\times 10^{7}$ cells per dish. We then replaced the media in each dish with 25 mL of WSN growth media in each dish containing 3.7 TCID${}_{50}$ of virus per μL. We allowed virus replication to proceed for 40 h before collecting viruses from the supernatant for sequencing.

### 4.4. Barcoded Subamplicon Sequencing

For each of the three replicate HA virus libraries and the wild-type HA virus, we extracted viral RNA by ultracentrifuging 24 mL of supernatant at 22,000 rpm in a Beckman Coulter SW28 rotor. RNA was extracted using the Qiagen RNeasy kit by resuspending the viral pellet in 400 μL buffer of Qiagen RLT freshly supplemented with *β*-mercaptoethanol, pipetting 30 times, transferring to an RNase-free microcentrifugefuge tube, adding 600 μL freshly-made 70% ethanol, and continuing with the manufacturer’s recommended protocol, eluting the final RNA product in 40 μL of RNase-free water. HA was then reverse transcribed using AccuScript Reverse Transcriptase (Agilent 200820) with the primers WSNHA-For (5’-AGCAAAAGCAGGGGAAAATAAAAACAAC-3’) and WSNHA-Rev (5’-AGTAGAAACAAGGGTGTTTTTCCTTATATTTCTG-3’).

We generated PCR amplicons of HA for each of the eight samples (three replicate plasmid DNA libraries, three corresponding virus libraries, one wild-type plasmid DNA, and one wild-type virus) using KOD Hot Start Master Mix (71842, EMD Millipore) with the PCR reaction mixture and cycling conditions described in [5] and the primers WSNHA-For and WSNHA-Rev. The templates for these reactions were 2 μL of cDNA (for the virus-derived samples) or 2 μL of plasmid DNA at 10 ng/μL. To ensure that the number of molecules used as template did not bottleneck diversity, parallel PCR reactions were run with a standard curve of template molecules, and all products were analyzed by band intensity after agarose gel electrophoresis; all samples used ≥10${}^{6}$ molecules as a template for PCR. We purified these PCR amplicons using Agencourt AMPure XP beads (bead-to-sample ratio 0.9) (Beckman Coulter).

These PCR amplicons were quantified using Quant-iT PicoGreen dsDNA Assay Kit (Life Technologies) and used as the templates for the barcoded-subamplicon sequencing in Figure 1B. We performed the first round of PCR (“PCR 1”) in six parallel reactions (one for each of the six HA subamplicons) for each of the eight samples. Each reaction contained 12 μL 2X KOD Hot Start Master Mix, 2 μL forward primer diluted to 5 μM, 2 μL reverse primer diluted to 5 μM, and 8 μL purified amplicon diluted to 0.5 ng/μL (primer sequences for PCR 1 and PCR 2 are provided in File S5). In addition to containing sequences targeting regions in HA, the forward and reverse primers for PCR 1 each contain an 8-base degenerate barcode and partial Illumina sequencing adaptors. To limit the generation of PCR artifacts, we performed only 9 cycles of PCR for PCR 1 using the following program: 1. 95 ${}^{\circ }$C for 2:00; 2. 95 ${}^{\circ }$C for 0:20; 3. 70 ${}^{\circ }$C for 0:01; 4. 54 ${}^{\circ }$C for 0:20; 5. 70 ${}^{\circ }$C for 0:20; 6. Go to 2 (8 times); 7. 95 ${}^{\circ }$C for 1:00; and 8. 4 ${}^{\circ }$C hold. The denaturation step after cycling ensures that identical barcode pairs are not annealed at the end, so that most double-stranded molecules entering PCR 2 will contain two unique barcoded mutants. PCR 1 products were purified by Ampure XP (bead-to-sample ratio 1.0), quantified with Quant-iT PicoGreen, and diluted to 0.5 ng/μL.

We then mixed all six subamplicons from each experimental sample at equal concentrations and diluted these subamplicon pools such that the number of template molecules used in PCR 2 was less than the anticipated sequencing depth to ensure multiple reads per barcode. Specifically, we reduced the total amount of DNA for each experimental sample used as template in PCR 2 to $9.24\times 10^{-4}$ ng, which corresponds to $1.54\times 10^{-4}$ ng of each of the six subamplicons, corresponding to approximately $3.5\times 10^{5}$ double-stranded DNA molecules (or $7\times 10^{5}$ uniquely-barcoded single-stranded variants) per subamplicon per sample.

We performed PCR 2 for each sample with the following reaction conditions: 20 uL 2X KOD Hot Start Master Mix, 4 μL forward primer UniversalRnd2 for diluted to 5 μM, 4 μL reverse primer indexXXRnd2rev diluted to 5 μM (a different index for each experimental sample), and $9.24\times 10^{-4}$ ng of the subamplicon pool of PCR 1 products described above, for a total volume of 40 μL. We used the following thermal cycling program: 1. 95 ${}^{\circ }$C for 2:00; 2. 95 ${}^{\circ }$C for 0:20; 3. 70 ${}^{\circ }$C for 0:01; 4. 55 ${}^{\circ }$C for 0:20; 5. 70 ${}^{\circ }$C for 0:20; 6. Go to 2 (23 times); and 7. 4 ${}^{\circ }$C hold. PCR 2 products were purified by Ampure XP (bead-to-sample ratio 1.0), quantified with Quant-iT PicoGreen, and equal amounts of each experimental sample were mixed and purified by agarose gel electrophoresis, excising the predominant DNA species at the expected size of approximately 470 bp. Sequencing was performed on one lane of a flow cell of an Illumina HiSeq 2500 using 2 × 250 bp paired-end reads in rapid-run mode.

### 4.5. Inference of Amino-Acid Preferences from Sequencing Data

We used dms_tools (http://jbloomlab.github.io/dms_tools/), version 1.1.12, to align subamplicon reads to a reference HA sequence, group barcodes to build consensus sequences, quantify mutation counts at every site in the gene for each experimental sample, and infer site-specific amino-acid preferences based on mutation frequencies pre- and post-selection using the algorithm described in [23]. The code that performs these analyses is in File S4.

### 4.6. Phylogenetic Modeling Using Amino-Acid Preferences

We sub-sampled human and swine H1 sequences (1 sequence per host per year) from the set of sequences from [4], removed identical sequences, and built a sequence alignment. We then used phydms version 1.1.0 [24] (http://jbloomlab.github.io/phydms/), which in turn uses Bio++ [25] for the likelihood calculations, to compare experimentally informed codon substitution models and other non-site-specific substitution models. The code that performs these analyses is in File S4.

### 4.7. Statistical Tests

Multiple linear regression of the continuous dependent variable of site entropy as a function of the continuous independent variable of relative solvent accessibility and a binary indicator of a site belonging to a specific classification (e.g., “antigenic sites”) was performed with the same classifications as described in [4]. Additional classifications were obtained from [34] for sites responsible for antigenic cluster transitions in H3N2 and seasonal H1N1 (sites 158, 168, 169, 171, 172, 202, and 206 in sequential WSN H1 numbering starting with the initiating methionine), and the sites within antibody footprints of broadly-neutralizing antibodies F10, CR6261, FI6v3, and CR9114 (sites 25, 45, 46, 47, 48, 49, 305, 306, 307, 332, 361, 362, 363, 364, 379, 381, 382, 384, 385, 386, 388, 389, 391, 392, 395, 396, 399, and 400 in sequential WSN H1 numbering starting with the initiating methionine) [35,36,37,38]. Definition of the protein domains within HA were from [39] (HA1 fusion domain: 18–72, 291–340; HA1 vestigial esterase domain: 73–125, 279–290; HA1 receptor binding domain: 126–278; HA2 fusion domain: 344–503; and all sites in sequential H1 numbering starting with the initiating methionine). The code that performs these analyses is in File S4.

### 4.8. Availability of Data and Computer Code

Sequencing data are available from the Sequence Read Archive under accession numbers SRR3113656 (mutant DNA library 1), SRR3113657 (mutant DNA library 2), SRR3113658 (mutant DNA library 3), SRR3113660 (mutant virus library 1), SRR3113661 (mutant virus library 2), SRR3113662 (mutant virus library 3), SRR3113655 (wild-type DNA control), and SRR3113659 (wild-type virus control). An iPython notebook (and a static HTML version of it) for all analyses is in File S4. A Python script for visualizing mutational tolerance on the HA structure in PyMol is in File S1.

## Figures and Tables

**Table 1:** **The site-specific amino-acid preferences measured in the new experiments offer an improved description of HA evolution in nature.** Aikake information criterion (AIC) [26] was used compare the maximum likelihood phylogenetic fit of several models to an alignment of seasonal human H1N1 and classical swine H1N1 HAs. The experimentally informed substitution models are of the form described in [24] with the data from the average of all three replicates of the new or old experiments, or the average of the two. These models are compared to the variants of the substitution model of Goldman *et al.* [27] denoted as M0 and M8 in Yang *et al.* [28] with the equilibrium codon frequencies estimated empirically using the F3X4 method. The best model is the one that combines all experimental data, but a model informed by the new experiments alone is better than one informed by the old experiments alone. To confirm that the experimentally informed models are superior because they are site specific, we fit a control model in which the experimental data is averaged across sites. The tree topology was fixed to that inferred by maximum likelihood using the M0 version of the Goldman–Yang model. The free parameters for each model were then optimized along with the branch lengths; optimized parameters are in the last column.

Model	ΔAIC	Log Likelihood	Parameters (Optimized + Empirical): Optimized Values
new data + old data	0.0	−14933.5	6 (6 + 0): *β* = 1.82, *ω* = 0.51, *κ* = 4.95, $\phi_{A}$ = 0.40, $\phi_{C}$ = 0.18, $\phi_{G}$ = 0.20
new data	197.6	−15032.3	6 (6 + 0): *β* = 1.80, *ω* = 0.46, *κ* = 5.06, $\phi_{A}$ = 0.40, $\phi_{C}$ = 0.18, $\phi_{G}$ = 0.20
old data	341.2	−15104.1	6 (6 + 0): *β* = 1.40, *ω* = 0.46, *κ* = 4.90, $\phi_{A}$ = 0.39, $\phi_{C}$ = 0.18, $\phi_{G}$ = 0.20
Goldman–Yang M8	2156.8	−16003.9	14 (5 + 9): $p_{\omega >1}$ = 0.01, $\omega_{>1}$ = 1.91, $p_{\beta }$ = 0.02, $q_{\beta }$ = 0.76, *κ* = 4.94
new data + old data, averaged across sites	2971.6	−16419.3	6 (6 + 0): *β* = 0.50, *ω* = 0.20, *κ* = 5.38, $\phi_{A}$ = 0.38, $\phi_{C}$ = 0.18, $\phi_{G}$ = 0.21
Goldman–Yang M0	2980.8	−16418.9	11 (2 + 9): *ω* = 0.19, *κ* = 4.88

## Supplementary Materials

The following figures are available online at www.mdpi.com/1999-4915/8/6/155/s1, and the following files are available online at www.mdpi.com/1999-4915/8/6/155/s2, **Figure S1**: An HA-deficient helper virus can replicate in cells constitutively expressing HA protein. **Figure S2**: The mutant plasmid DNA library used in this study (“new”) has a lower mutation rate than the library used by [4] (“old”). **Figure S3**: Mutant virus library generation is more efficient when HA is encoded on the pHH21 plasmid. **Figure S4**: Purging of stop codons is more complete in our new experiment than in the previous one. **Figure S5**: Synonymous frequency peaks observed in bottlenecked virus libraries are not due to the composition of plasmid mutant libraries. **Figure S6**: Statistical analyses of whether sets of sites have higher or lower mutational tolerance than expected given their solvent accessibility. **Figure S7**: The mutational tolerance of HA’s domains. **File S1**: PyMol script for visualization of mutational tolerance on the HA crystal structure. **File S2**: Text file with the overall merged site-specific amino-acid preferences (average of the new and old data). Residues are numbered sequentially beginning with 1 at the N-terminal methionine. Conversion between WSN sequential numbering and H3 numbering is provided in File S6. **File S3**: Text file with the overall merged site-specific amino-acid preferences (average of the new and old data) scaled by the stringency parameter from Table 1. Residues are numbered sequentially beginning with 1 at the N-terminal methionine. Conversion between WSN sequential numbering and H3 numbering is provided in File S6. **File S4**: A ZIP /hlfile containing the data and code for all data analysis and figure generation, **File S5**: Text file with primer sequences for the barcoded subamplicon sequencing, **File S6**: Text file with HA site numbering conversion between H3 numbering and sequential numbering of the WSN HA beginning with 1 at the N-terminal methionine.

## Abbreviations

The following abbreviations are used in this manuscript:

HA: hemagglutinin
MOI: multiplicity of infection
TCID_50_: 50% tissue culture infectious dose
