# Supplementary material for: Accurate Measurement of the Effects of All Amino-Acid Mutations on Influenza Hemagglutinin
Source: Viruses. 2016 Jun 3;8(6):155. doi: 10.3390/v8060155 (PMC4926175; doi:10.3390/v8060155)
Supplement: Supplementary file 1 [file viruses-08-00155-s001.pdf]

Supplementary Materials: Accurate Measurement of the Effects of All Amino-Acid Mutations on Influenza Hemagglutinin

Michael B. Doud and Jesse D. Bloom

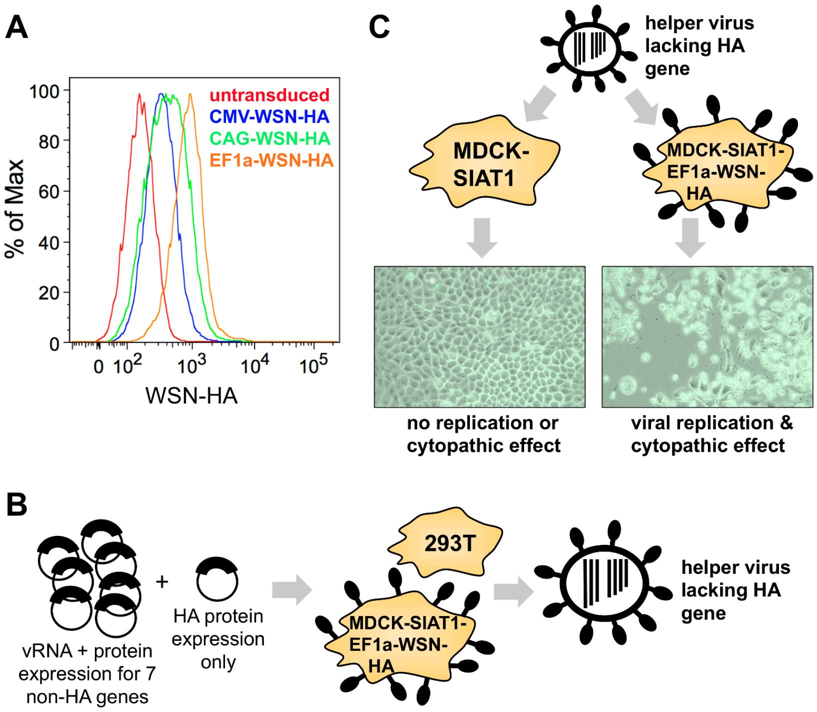

Figure S1. An HA-deficient helper virus can replicate in cells constitutively expressing HA protein.

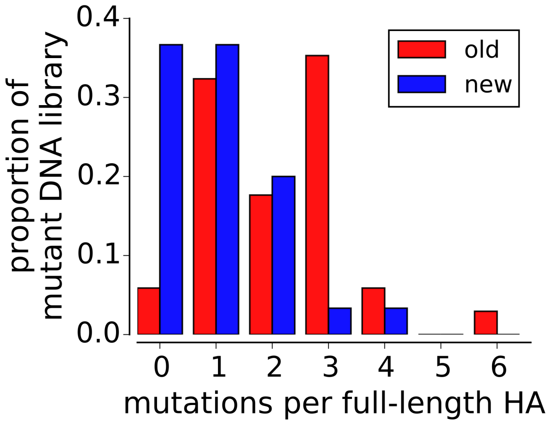

Figure S2. The mutant plasmid DNA library used in this study ("new") has a lower mutation rate than the library used by [4] ("old").

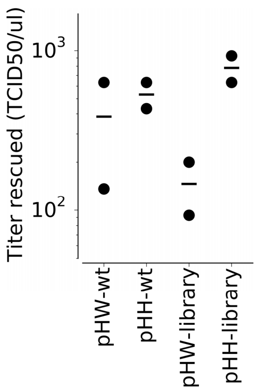

Figure S3. Mutant virus library generation is more efficient when HA is encoded on the pHH21 plasmid.

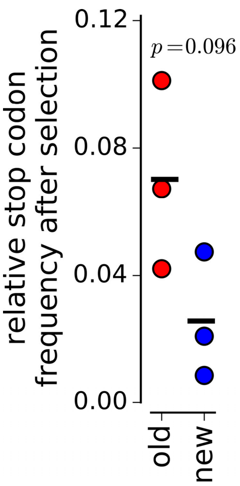

Figure S4. Purging of stop codons is more complete in our new experiment than in the previous one.

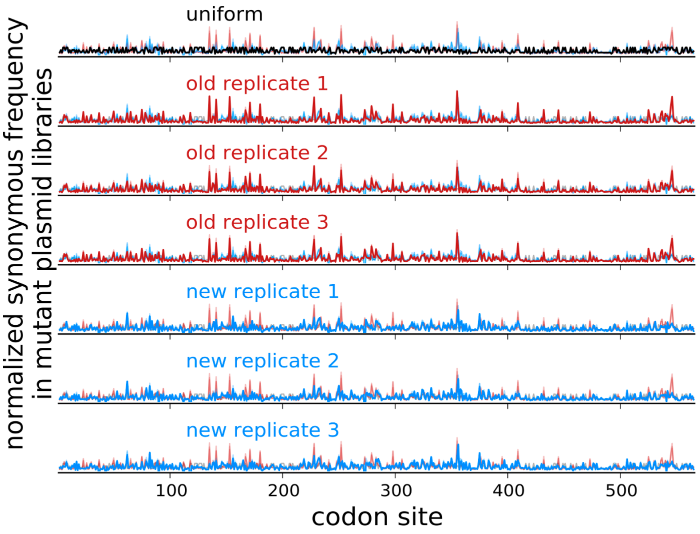

Figure S5. Synonymous frequency peaks observed in bottlenecked virus libraries are not due to the composition of plasmid mutant libraries.

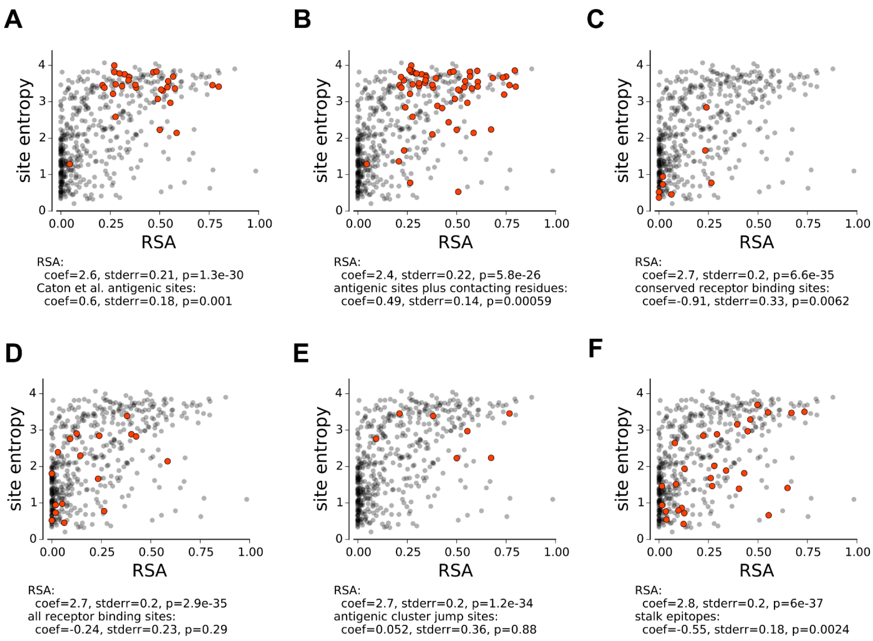

Figure S6. Statistical analyses of whether sets of sites have higher or lower mutational tolerance than expected given their solvent accessibility.

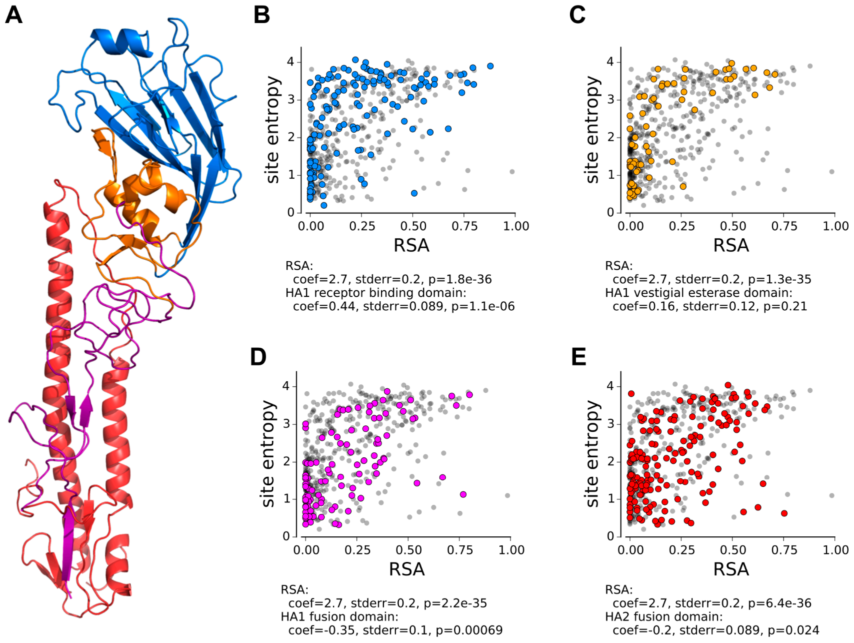

Figure S7. The mutational tolerance of HA's domains.

© 2016 by the authors, licensee MDPI, Basel, Swizerland. This article is an open access article distributed under the terms and conditions of the Creative Commons Attribution (CC-BY) license (http://creativecommons.org/licenses/by/4.0/).
