# Supplementary material for: Accurate Measurement of the Effects of All Amino-Acid Mutations on Influenza Hemagglutinin
Source: Viruses. 2016 Jun 3;8(6):155. doi: 10.3390/v8060155 (PMC4926175; doi:10.3390/v8060155)
Supplement: Supplementary file 2 [file viruses-08-00155-s002.zip › Supplemental_File_4_analysiscode.zip!/Supplemental_File_4_analysiscode/AnalysisNotebook.html]

AnalysisNotebook


# Analysis of deep mutational scanning of influenza A/WSN/1933(H1N1) hemagglutinin¶

Mike Doud, 20 May 2016

---

## Table of contents¶

### Load required modules and raw data¶

Preliminary set-up and load required modules

Download sequencing data from the SRA

Download data from Thyagarajan and Bloom, 2014

### Process and analyze data¶

Alignment, mutation parsing, and inference of amino-acid preferences with \*dms\_tools\*

Phylogenetic analysis

### Make figures¶

Mutation fraction breakdown across samples

Purging of stop codon mutants during selection

Synonymous codon frequencies across sites in all replicates

Amino-acid preference correlations between experimental replicates

Model site entropy as a function of relative solvent accessibility

---

## Load required modules and raw data¶

**Preliminary set-up and load required modules:**

In [1]:

```
import os
base_directory = os.getcwd()
if not os.path.isdir('notebook_figures'):
    !mkdir notebook_figures
if not os.path.isdir('FASTQ_files'):
    !mkdir FASTQ_files
if not os.path.isdir('Thyagarajan2014'):
    !mkdir Thyagarajan2014
if not os.path.isdir('preferences'):
    !mkdir preferences
if not os.path.isdir('phylogenetics'):
    !mkdir phylogenetics

from runscriptfunctions import * # functions to facilitate running parallel jobs on the Fred Hutch scientific computing cluster using `slurm`
from alignmentfunctions import * # functions to assist in building a sequence alignment of H1 for phylogenetic analysis
import matplotlib
print "Using matplotlib version %s" % matplotlib.__version__
matplotlib.use("Pdf")
orange = "#E69F00"
lightblue = "#56B4E9"
green = "#009E73"
yellow = "#F0E442"
darkblue = "#0072B2"
rust =  "#D55E00"
purple =  "#CC79A7"
from matplotlib.ticker import MaxNLocator
from matplotlib.patches import Polygon
from matplotlib import gridspec
import pylab as plt
from IPython.display import Image, display
import dms_tools
print "Using dms_tools version %s" % dms_tools.__version__
import dms_tools.utils
import dms_tools.file_io
import dms_tools.plot
import mapmuts.dssp
import glob
import pandas as pd
import scipy.stats
import numpy as np
import statsmodels
import statsmodels.api as sm
import statsmodels.formula.api as smf
print "Using statsmodels version %s" % statsmodels.__version__
!phydms -v
```

```
Using matplotlib version 1.4.2
Using dms_tools version 1.1.12
Using statsmodels version 0.6.1
phydms 1.1.0
```

**Download sequencing data from the SRA:**

In [ ]:

```
%%capture
samples = {
    'SRR3113656':'mutDNA1',
    'SRR3113657':'mutDNA2',
    'SRR3113658':'mutDNA3',
    'SRR3113660':'mutvirus1',
    'SRR3113661':'mutvirus2',
    'SRR3113662':'mutvirus3',
    'SRR3113655':'wtDNA',
    'SRR3113659':'wtvirus'}
for (accession, samplename) in samples.items():
    print "Downloading and converting file format for sample %s, which corresponds to accession %s." % (samplename, accession)
    !cd FASTQ_files; wget --no-clobber 'ftp://ftp-trace.ncbi.nih.gov/sra/sra-instant/reads/ByRun/sra/SRR/SRR311/'$accession'/'$accession'.sra'
    !mv "FASTQ_files/"$accession".sra" "FASTQ_files/"$samplename".sra"
    !fastq-dump -O FASTQ_files/ --split-files "FASTQ_files/"$samplename".sra"
```

**Download data from Thyagarajan and Bloom, 2014:**

In [ ]:

```
%%capture
# Download the inferred amino-acid preferences as well as the codon counts files summarizing the deep sequencing data
for replicate in [1,2,3]:
    !cd Thyagarajan2014; wget --no-clobber 'https://raw.githubusercontent.com/jbloom/mapmuts/master/examples/WSN_HA_2014Analysis/replicate_'$replicate'/replicate_'$replicate'_equilibriumpreferences.txt'
    !cd Thyagarajan2014; mv 'replicate_'$replicate'_equilibriumpreferences.txt' 'Thyagarajan2014-rep'$replicate'_prefs.txt'
    for sample in ['DNA','virus','mutDNA','mutvirus']:
        countsfile = 'https://raw.githubusercontent.com/jbloom/mapmuts/master/examples/WSN_HA_2014Analysis/replicate_%s/%s/replicate_%s_%s_codoncounts.txt' % (replicate,sample,replicate,sample)
        !cd Thyagarajan2014; wget --no-clobber $countsfile
# Download the same site classification files that were used in (Thyagarajan and Bloom, 2014) for Caton sites, Caton sites + contacting sites, 
# all receptor binding sites, and conserved receptor binding sites.
!cd Thyagarajan2014; wget --no-clobber 'https://raw.githubusercontent.com/jbloom/mapmuts/master/examples/WSN_HA_2014Analysis/Caton_H1_HA_antigenic_sites.txt'
!cd Thyagarajan2014; wget --no-clobber 'https://raw.githubusercontent.com/jbloom/mapmuts/master/examples/WSN_HA_2014Analysis/nearby_antigenic_sites.txt'
!cd Thyagarajan2014; wget --no-clobber 'https://raw.githubusercontent.com/jbloom/mapmuts/master/examples/WSN_HA_2014Analysis/allRBS_residues.txt'
!cd Thyagarajan2014; wget --no-clobber 'https://raw.githubusercontent.com/jbloom/mapmuts/master/examples/WSN_HA_2014Analysis/receptor_binding_residues.txt'
# Download the H1 sequences sampled to build the alignment used for phylogenetic analysis in (Thyagarajan and Bloom, 2014):
!cd phylogenetics; wget --no-clobber 'https://raw.githubusercontent.com/jbloom/phyloExpCM/master/examples/2014Analysis_Influenza_H1_HA/H1_HAseqs.fasta'
!cd phylogenetics; wget --no-clobber 'https://raw.githubusercontent.com/jbloom/phyloExpCM/master/examples/2014Analysis_Influenza_H1_HA/JVI_82_8947_Anomalies.txt'
!cd phylogenetics; wget --no-clobber 'https://raw.githubusercontent.com/jbloom/phyloExpCM/master/examples/2014Analysis_Influenza_H1_HA/JDB_Anomalies.txt'
```

---

## Process and analyze data¶

**Alignment, mutation parsing, and inference of amino-acid preferences with *dms\_tools*:**

In [ ]:

```
%%writefile WSN_HA_reference.fa
>Reference_WSN_HA_coding_sequence
atgaaggcaaaactactggtcctgttatatgcatttgtagctacagatgcagacacaatatgtataggctaccatgcgaacaactcaaccgacactgttgacacaatactcgagaagaatgtggcagtgacacattctgttaacctgctcgaagacagccacaacgggaaactatgtaaattaaaaggaatagccccactacaattggggaaatgtaacatcaccggatggctcttgggaaatccagaatgcgactcactgcttccagcgagatcatggtcctacattgtagaaacaccaaactctgagaatggagcatgttatccaggagatctcatcgactatgaggaactgagggagcaattgagctcagtatcatcattagaaagattcgaaatatttcccaaggaaagttcatggcccaaccacacattcaacggagtaacagtatcatgctcccataggggaaaaagcagtttttacagaaatttgctatggctgacgaagaagggggattcatacccaaagctgaccaattcctatgtgaacaataaagggaaagaagtccttgtactatggggtgttcatcacccgtctagcagtgatgagcaacagagtctctatagtaatggaaatgcttatgtctctgtagcgtcttcaaattataacaggagattcaccccggaaatagctgcaaggcccaaagtaagagatcaacatgggaggatgaactattactggaccttgctagaacccggagacacaataatatttgaggcaactggtaatctaatagcaccatggtatgctttcgcactgagtagagggtttgagtccggcatcatcacctcaaacgcgtcaatgcatgagtgtaacacgaagtgtcaaacaccccagggagctataaacagcaatctccctttccagaatatacacccagtcacaataggagagtgcccaaaatatgtcaggagtaccaaattgaggatggttacaggactaagaaacatcccatccattcaatacagaggtctatttggagccattgctggttttattgaggggggatggactggaatgatagatggatggtatggttatcatcatcagaatgaacagggatcaggctatgcagcggatcaaaaaagcacacaaaatgccattaacgggattacaaacaaggtgaactctgttatcgagaaaatgaacactcaattcacagctgtgggtaaagaattcaacaacttagaaaaaaggatggaaaatttaaataaaaaagttgatgatgggtttctggacatttggacatataatgcagaattgttagttctactggaaaatgaaaggactttggatttccatgacttaaatgtgaagaatctgtacgagaaagtaaaaagccaattaaagaataatgccaaagaaatcggaaatgggtgttttgagttctaccacaagtgtgacaatgaatgcatggaaagtgtaagaaatgggacttatgattatccaaaatattcagaagaatcaaagttgaacagggaaaagatagatggagtgaaattggaatcaatgggggtgtatcagattctggcgatctactcaactgtcgccagttcactggtgcttttggtctccctgggggcaatcagtttctggatgtgttctaatgggtctttgcagtgcagaatatgcatctga
```

In [ ]:

```
# set some parameters and organize the fastq file paths
use_sbatch = True
max_cpus = 64
seed = 1
refseq = '%s/WSN_HA_reference.fa' % base_directory
samples = ['wtDNA','wtvirus','mutDNA1','mutDNA2','mutDNA3','mutvirus1','mutvirus2','mutvirus3']
alignspecs = '1,285,36,37 286,570,31,32 571,855,37,32 856,1140,31,36 1141,1425,29,33 1426,1698,40,43'
fastqpath = '%s/FASTQ_files/' % base_directory
fastqfiles = {} # keyed by sample, values (r1files, r2files)
for sample in samples:
    r1files = sorted(glob.glob('%s/%s_1.fastq' % (fastqpath, sample)))
    r2files = sorted(glob.glob('%s/%s_2.fastq' % (fastqpath, sample)))
    assert r1files and r2files
    fastqfiles[sample] = (r1files, r2files)
```

In [ ]:

```
%%capture
# make the alignments and parse mutation counts to be written to *counts.txt files:
run_directory = 'alignments/'
processes = []
alignprefixes_names = []
for sample in samples:
    alignprefix = sample
    countsfile = '%scounts.txt' % alignprefix
    statsfile = '%ssummarystats.txt' % alignprefix
    (r1files, r2files) = fastqfiles[sample]
    commands = ['%s %s %s %s %s' % (alignprefix, refseq, ','.join(r1files), ','.join(r2files), alignspecs), '--R1trimlength 200', '--R2trimlength 170', '--barcodeinfo']
    processes.append(multiprocessing.Process(target=RunScript,\
        args=(run_directory, 'align_%s' % (sample), 'dms_barcodedsubamplicons', commands, use_sbatch, 4),
        ))
    alignprefixes_names.append("%s,%s" % (alignprefix, sample.replace('_','')))
RunProcesses(processes, nmultiruns=max_cpus)
```

In [ ]:

```
%%capture
# summarize the alignments
run_directory = 'alignments/'
processes = []
for replicate in [1,2,3]:
    outprefix = 'rep_%s_alignmentsummary' % (replicate)
    these_alignprefixes_names = [   'wtDNA,wtDNA', 'wtvirus,wtvirus', 'mutDNA%s,mutDNA%s'%(replicate,replicate),
                                    'mutvirus%s,mutvirus%s'%(replicate,replicate) ]
    commands = ['%s barcodedsubamplicons %s --writemutfreqs' % (outprefix, ' '.join(these_alignprefixes_names))]
    processes.append(multiprocessing.Process(target=RunScript,\
        args=(run_directory, 'rep%s_summarize' % (replicate), 'dms_summarizealignments', commands, use_sbatch, 1),
        ))
RunProcesses(processes, nmultiruns=max_cpus)
```

Calculate fraction of possible mutations present in the plasmid mutant libraries on the codon and amino-acid levels:

In [27]:

```
def num_mismatches(codon1, codon2):
    mm = 0
    for nt1, nt2 in zip(codon1, codon2):
        if nt1 != nt2:
            mm += 1
    return mm

def possible_mutations(codon, muttype, only_multi_nt = False):
    '''Returns a list of possible codon mutations of the given *muttype* ('NS', 'SYN' or 'ALL')
    for a given *codon*. If only_multi_nt is True, only returns multinucleotide mutations.
    if muttype is 'AA', returns possible amino acid mutations, and only_multi_nt cannot be used.
    '''
    wt_aa = dms_tools.codon_to_aa[codon]
    if muttype == 'NS':
        muts = [c for c in dms_tools.codons if dms_tools.codon_to_aa[c] != wt_aa]
    elif muttype == 'SYN':
        muts = [c for c in dms_tools.codons if dms_tools.codon_to_aa[c] == wt_aa and c != codon]
    elif muttype == 'ALL':
        muts = [c for c in dms_tools.codons if c != codon]
    elif muttype == 'AA':
        muts = [a for a in dms_tools.aminoacids_nostop if a != wt_aa]
    else:
        raise ValueError("Not an allowed muttype: %s" % muttype)
    if only_multi_nt:
        assert muttype != 'AA'
        return [c for c in muts if num_mismatches(c,codon) > 1]
    else:
        return muts

def TallyMutationsAboveThresholds(filename, bins, muttype, sites, counts_or_freqs = 'counts', only_multi_nt = False):
    '''Calculates the fraction of all mutations of *muttype* ('SYN', 'NS', or 'ALL') that appear greater than or
    equal to the *counts_or_freqs* ('counts' or 'freqs') for the threshold values listed in *bins*.
    
    Also possible to use 'AA' to analyze on the amino-acid mutation level, instead of the codon level. If so, only_multi_nt cannot be used.
    
    Only considers mutations for the sites listed in *sites*.
    
    For example, if *bins* is [1,2,3,4,5,6,7,8] and *counts_or_freqs* = 'counts', this would
    return a list of eight values, each being the fraction of mutations observed greater or 
    equal to once, twice, three times, four times, etc.
    '''
    assert muttype in ['SYN', 'NS', 'ALL', 'AA']
    assert counts_or_freqs in ['counts', 'freqs']
    sites = [str(s) for s in sites]
    counts = dms_tools.utils.ClassifyCodonCounts(dms_tools.file_io.ReadDMSCounts(filename, "codon"))
    total_possible_muts = 0 # across entire gene.
    bin_counts = [0 for _ in bins] # placeholders for tallying number of mutations falling into each bin
    for site in sites:
        wt_codon = counts[site]['WT']
        muts = possible_mutations(wt_codon, muttype, only_multi_nt = only_multi_nt) # can be either codons or AAs.
        total_possible_muts += len(muts) # tally total number of possible codon or aa muts across all sites.
        
        for mut in muts:
            
            if muttype == 'AA':
                aa_counts_of_this_mut = 0
                for codon_i in dms_tools.aa_to_codons[mut]:
                    aa_counts_of_this_mut += counts[site][codon_i]
                if counts_or_freqs == 'counts':
                    mut_abundance = aa_counts_of_this_mut
                elif counts_or_freqs == 'freqs':
                    mut_abundance = float(aa_counts_of_this_mut)/counts[site]['COUNTS']
            else: # else process on codon level:
                if counts_or_freqs == 'counts':
                    mut_abundance = counts[site][mut]
                elif counts_or_freqs == 'freqs':
                    mut_abundance = float(counts[site][mut])/counts[site]['COUNTS']
                    
            for i, binvalue in enumerate(bins):
                if mut_abundance >= binvalue:
                    bin_counts[i] += 1
    # now normalize bin_counts to the total number of possible mutations so that
    # these values represent the fraction of possible mutations
    bin_counts = [float(num)/total_possible_muts for num in bin_counts]
    return bin_counts

mut_plasmid_counts_files = ['alignments/mutDNA%scounts.txt' % i for i in [1,2,3]]
sites = range(2,566)

bins = [1]
muttype = 'ALL'
for f in mut_plasmid_counts_files:
    print "fraction of all codon mutations observed >= %s times in %s: %.4f" % (bins[0],f, TallyMutationsAboveThresholds(f,bins,muttype,sites,only_multi_nt=False)[0])
    
bins = [1]
muttype = 'AA'
for f in mut_plasmid_counts_files:
    print "fraction of all amino-acid mutations observed >= %s times in %s: %.4f" % (bins[0],f, TallyMutationsAboveThresholds(f,bins,muttype,sites,only_multi_nt=False)[0])
    
bins = [3]
muttype = 'AA'
for f in mut_plasmid_counts_files:
    print "fraction of all amino-acid mutations observed >= %s times in %s: %.4f" % (bins[0],f,TallyMutationsAboveThresholds(f,bins,muttype,sites,only_multi_nt=False)[0])
    
bins = [5]
muttype = 'AA'
for f in mut_plasmid_counts_files:
    print "fraction of all amino-acid mutations observed >= %s times in %s: %.4f" % (bins[0],f, TallyMutationsAboveThresholds(f,bins,muttype,sites,only_multi_nt=False)[0])
```

```
fraction of all codon mutations observed >= 1 times in alignments/mutDNA1counts.txt: 0.9814
fraction of all codon mutations observed >= 1 times in alignments/mutDNA2counts.txt: 0.9881
fraction of all codon mutations observed >= 1 times in alignments/mutDNA3counts.txt: 0.9760
fraction of all amino-acid mutations observed >= 1 times in alignments/mutDNA1counts.txt: 0.9957
fraction of all amino-acid mutations observed >= 1 times in alignments/mutDNA2counts.txt: 0.9966
fraction of all amino-acid mutations observed >= 1 times in alignments/mutDNA3counts.txt: 0.9946
fraction of all amino-acid mutations observed >= 3 times in alignments/mutDNA1counts.txt: 0.9797
fraction of all amino-acid mutations observed >= 3 times in alignments/mutDNA2counts.txt: 0.9870
fraction of all amino-acid mutations observed >= 3 times in alignments/mutDNA3counts.txt: 0.9753
fraction of all amino-acid mutations observed >= 5 times in alignments/mutDNA1counts.txt: 0.9512
fraction of all amino-acid mutations observed >= 5 times in alignments/mutDNA2counts.txt: 0.9707
fraction of all amino-acid mutations observed >= 5 times in alignments/mutDNA3counts.txt: 0.9378
```

In [ ]:

```
%%capture
# infer preferences for the three replicate experiments
run_directory = 'preferences/'
wtDNA_file = '%s/alignments/wtDNAcounts.txt' % base_directory
wtvirus_file = '%s/alignments/wtviruscounts.txt' % base_directory
processes = []
for replicate in [1,2,3]:
    prefs = '2016-WSNHA-rep%d_prefs.txt' % (replicate)
    mutDNA_file = '%s/alignments/mutDNA%scounts.txt' % (base_directory, replicate)
    mutvirus_file = '%s/alignments/mutvirus%scounts.txt' % (base_directory, replicate)
    commands = ['%s %s %s' % (mutDNA_file, mutvirus_file, prefs), '--errpre %s' % wtDNA_file, '--errpost %s' % wtvirus_file, '--ncpus -1']
    processes.append(multiprocessing.Process(target=RunScript,\
            args=(run_directory, 'rep%s_prefs' % (replicate), 'dms_inferprefs', commands, use_sbatch, 12) ))
RunProcesses(processes, nmultiruns=max_cpus)
```

In [ ]:

```
%%writefile trimsites.txt
#Trim sites 1 and 566, the start and stop codons, from preferences files before phylogenetic analysis
1
566
```

In [ ]:

```
%%capture
# process the preferences files for phylogenetic analysis by excluding stop preferences, trimming start and stop sites, and averaging across replicates.
# do for both the new data processed here, as well as the preferences from Thyagarajan and Bloom, 2014
trimfile = '%s/trimsites.txt' % base_directory

# process new preference data
run_directory = 'preferences/'
for rep in [1,2,3]: 
    raw_prefs = '2016-WSNHA-rep%d_prefs.txt' % rep
    exclude_stop_prefs = raw_prefs.replace('.txt', '_excludestop.txt')
    commands = ['%s average %s --excludestop' % (exclude_stop_prefs, raw_prefs)]
    RunScript(run_directory, 'exclude_rep%s' % rep, 'dms_merge', commands, False, 1)
    trimmed_prefs = raw_prefs.replace('.txt', '_trimmed.txt')
    commands = ['%s %s remove %s' % (exclude_stop_prefs, trimmed_prefs, trimfile)]
    RunScript(run_directory, 'trim_rep%s' % rep, 'dms_editsites', commands, False, 1)
files_to_merge = ['2016-WSNHA-rep%d_prefs_trimmed.txt' % rep for rep in [1,2,3]]
merged_prefs = '2016-WSNHA_merged_prefs.txt'
commands = ['%s average %s' % (merged_prefs, ' '.join(files_to_merge))]
RunScript(run_directory, 'merge2016', 'dms_merge', commands, False, 1)

# process Thyagarajan 2014 data
run_directory = 'Thyagarajan2014/'
for rep in [1,2,3]: 
    raw_prefs = 'Thyagarajan2014-rep%s_prefs.txt' % rep
    exclude_stop_prefs = raw_prefs.replace('.txt', '_excludestop.txt')
    commands = ['%s average %s --excludestop' % (exclude_stop_prefs, raw_prefs)]
    RunScript(run_directory, 'exclude_rep%s' % rep, 'dms_merge', commands, False, 1)
    trimmed_prefs = raw_prefs.replace('.txt', '_trimmed.txt')
    commands = ['%s %s remove %s' % (exclude_stop_prefs, trimmed_prefs, trimfile)]
    RunScript(run_directory, 'trim_rep%s' % rep, 'dms_editsites', commands, False, 1)
files_to_merge = ['Thyagarajan2014-rep%s_prefs_trimmed.txt' % rep for rep in [1,2,3]]
merged_prefs = 'Thyagarajan2014-WSNHA_merged_prefs.txt'
commands = ['%s average %s' % (merged_prefs, ' '.join(files_to_merge))]
RunScript(run_directory, 'merge2014', 'dms_merge', commands, False, 1)

# take average across the two studies and save it in preferences/
run_directory = 'preferences/'
files_to_merge = ['%s/preferences/2016-WSNHA_merged_prefs.txt' % base_directory, '%s/Thyagarajan2014/Thyagarajan2014-WSNHA_merged_prefs.txt' % base_directory]
merged_prefs = 'Overall-WSNHA_merged_prefs.txt'
commands = ['%s average %s' % (merged_prefs, ' '.join(files_to_merge))]
RunScript(run_directory, 'overallmerge', 'dms_merge', commands, False, 1)
```

`phydms` requires that the preferences files used as experimental codon models (ExpCM) begin with the first site numbered "1". Since we only have preferences for sites beginning with site 2 in the protein, we will now make preference files renumbered so that the true site 2 of the protein is labeled site 1 in the preferences file. These files will all have the suffix `_renumbered` appended to them.

In [12]:

```
# make a renumbering file for dms_editsites. This file lists on each line the old number, a space, and the new number:
f = open('%s/renumbering_scheme.txt' % base_directory, 'w')
f.write("#original_site new_site\n")
for original_number in range(2,566):
    f.write("%s %s\n" % (original_number, original_number-1))
f.close()
```

In [ ]:

```
%%capture
# now make renumbered files using dms_editsites:
ExpCM_models_torenumber =   ['preferences/2016-WSNHA-rep%s_prefs_trimmed.txt' % i for i in [1,2,3]] + ['preferences/2016-WSNHA_merged_prefs.txt'] + \
                            ['preferences/Overall-WSNHA_merged_prefs.txt'] + \
                            ['Thyagarajan2014/Thyagarajan2014-rep%s_prefs_trimmed.txt' % i for i in [1,2,3]] + ['Thyagarajan2014/Thyagarajan2014-WSNHA_merged_prefs.txt']

for f_to_renumber in ExpCM_models_torenumber:
    full_path_to_renumber = "%s/%s" % (base_directory, f_to_renumber)
    renumbered_file = full_path_to_renumber.replace(".txt", "_renumbered.txt")
    if "preferences/" in full_path_to_renumber:
        run_directory = 'preferences/'
    elif "Thyagarajan2014/" in full_path_to_renumber:
        run_directory = "Thyagarajan2014/"
    commands = ['%s %s renumber %s/renumbering_scheme.txt' % (full_path_to_renumber, renumbered_file, base_directory)]
    RunScript(run_directory, 'renumber', 'dms_editsites', commands, False, 1)
```

**Phylogenetic analysis:**

Make a sequence alignment with which to build a tree and test preference datasets as experimental codon substitution models.

This sequence alignment will be very similar to the one used in (Thyagarajan and Bloom, 2014), except that it will be a smaller sample (only 1 sequence per year per host) and the start codon will be removed from the sequences, since our experiments do not measure meangingful preferences at the start codon. This sequence alignment samples H1 sequences from human and swine lineages using the same set of all H1 sequences used in (Thyagarajan and Bloom, 2014), found in `phylogenetics/H1_HAseqs.fasta`. After removing anomolous sequences listed in (Krasnitz et al, 2008) or those that strongly violate the molecular clock, 1 sequence per host per year is retained, and then any remaining identical sequences are removed.

In [22]:

```
%%writefile phylogenetics/WSN_HA_reference_noStart_noStop.fa
>Reference_WSN_HA_coding_sequence_noStart_noStop
aaggcaaaactactggtcctgttatatgcatttgtagctacagatgcagacacaatatgtataggctaccatgcgaacaactcaaccgacactgttgacacaatactcgagaagaatgtggcagtgacacattctgttaacctgctcgaagacagccacaacgggaaactatgtaaattaaaaggaatagccccactacaattggggaaatgtaacatcaccggatggctcttgggaaatccagaatgcgactcactgcttccagcgagatcatggtcctacattgtagaaacaccaaactctgagaatggagcatgttatccaggagatctcatcgactatgaggaactgagggagcaattgagctcagtatcatcattagaaagattcgaaatatttcccaaggaaagttcatggcccaaccacacattcaacggagtaacagtatcatgctcccataggggaaaaagcagtttttacagaaatttgctatggctgacgaagaagggggattcatacccaaagctgaccaattcctatgtgaacaataaagggaaagaagtccttgtactatggggtgttcatcacccgtctagcagtgatgagcaacagagtctctatagtaatggaaatgcttatgtctctgtagcgtcttcaaattataacaggagattcaccccggaaatagctgcaaggcccaaagtaagagatcaacatgggaggatgaactattactggaccttgctagaacccggagacacaataatatttgaggcaactggtaatctaatagcaccatggtatgctttcgcactgagtagagggtttgagtccggcatcatcacctcaaacgcgtcaatgcatgagtgtaacacgaagtgtcaaacaccccagggagctataaacagcaatctccctttccagaatatacacccagtcacaataggagagtgcccaaaatatgtcaggagtaccaaattgaggatggttacaggactaagaaacatcccatccattcaatacagaggtctatttggagccattgctggttttattgaggggggatggactggaatgatagatggatggtatggttatcatcatcagaatgaacagggatcaggctatgcagcggatcaaaaaagcacacaaaatgccattaacgggattacaaacaaggtgaactctgttatcgagaaaatgaacactcaattcacagctgtgggtaaagaattcaacaacttagaaaaaaggatggaaaatttaaataaaaaagttgatgatgggtttctggacatttggacatataatgcagaattgttagttctactggaaaatgaaaggactttggatttccatgacttaaatgtgaagaatctgtacgagaaagtaaaaagccaattaaagaataatgccaaagaaatcggaaatgggtgttttgagttctaccacaagtgtgacaatgaatgcatggaaagtgtaagaaatgggacttatgattatccaaaatattcagaagaatcaaagttgaacagggaaaagatagatggagtgaaattggaatcaatgggggtgtatcagattctggcgatctactcaactgtcgccagttcactggtgcttttggtctccctgggggcaatcagtttctggatgtgttctaatgggtctttgcagtgcagaatatgcatc
```

```
Writing phylogenetics/WSN_HA_reference_noStart_noStop.fa
```

In [23]:

```
# this uses the MakeAlignment() function from alignmentfunctions.py, which is hardcoded to make an H1 alignment of human and swine sequences.
MakeAlignment('phylogenetics/H1_HAseqs.fasta', 1, 'phylogenetics/WSN_HA_reference_noStart_noStop.fa', 'phylogenetics/H1_1prYearHostSubtype_alignment.fasta', base_directory)
```

```
Using a reference sequence of Reference_WSN_HA_coding_sequence_noStart_noStop.
This sequence is 1692 nucleotides in length, and encodes a protein of 564 residues.

Read 14847 sequences from phylogenetics/H1_HAseqs.fasta.

Purging pre-defined anomalous sequences...
Retained 14295 sequences after removing anomalous ones.

Purging sequences that are not at least 1695 nucleotides long...
Retained 14292 sequences that are at least 1695 nucleotides long.

Purging any sequences with ambiguous nucleotide identities.
Retained 13195 sequences after purging those with ambiguous nucleotides.

Making sure the host / year / subtype can be parsed...
Retained 13063 sequences for which this information could be parsed.

Only keep sequences of H1N\d{1,2} subtype...
Retained 13063 sequences of subtype H1N\d{1,2}.

Parsing sequences by host and year...
For host Swine, retained following subtypes:
  H1N1: 1296 total sequences from 42 different years.
For host Human, retained following subtypes:
  H1N1: 10396 total sequences from 54 different years.
Overall, 11692 of the 13063 sequences were retained as an appropriate host / subtype / year.

Now retaining just 1 sequences per host / subtype / year, retaining those with the highest counts of that exact sequence for each year...
Retained 96 total sequences.
Removing redundant sequencies...
Retained 93 sequences after removing any identical sequences.

Now translating and aligning...
Successfully aligned all sequences.

Writing the aligned coding DNA sequences to phylogenetics/H1_1prYearHostSubtype_alignment.fasta.
```

Define the preferences-based models to be tested (all three replicates of each experiment, the merged preferences for each experiment, and the overall average across both experiments):

In [4]:

```
ExpCM_models =  ['preferences/2016-WSNHA-rep%s_prefs_trimmed_renumbered.txt' % i for i in [1,2,3]] + ['preferences/2016-WSNHA_merged_prefs_renumbered.txt'] + \
                ['preferences/Overall-WSNHA_merged_prefs_renumbered.txt'] + \
                ['Thyagarajan2014/Thyagarajan2014-rep%s_prefs_trimmed_renumbered.txt' % i for i in [1,2,3]] + ['Thyagarajan2014/Thyagarajan2014-WSNHA_merged_prefs_renumbered.txt']
        
print "These will be the preferences-based experimental codon models tested:"
for m in ExpCM_models:
    print m

# define full path for each
ExpCM_models = ['%s/%s' % (base_directory, f) for f in ExpCM_models]
```

```
These will be the preferences-based experimental codon models tested:
preferences/2016-WSNHA-rep1_prefs_trimmed_renumbered.txt
preferences/2016-WSNHA-rep2_prefs_trimmed_renumbered.txt
preferences/2016-WSNHA-rep3_prefs_trimmed_renumbered.txt
preferences/2016-WSNHA_merged_prefs_renumbered.txt
preferences/Overall-WSNHA_merged_prefs_renumbered.txt
Thyagarajan2014/Thyagarajan2014-rep1_prefs_trimmed_renumbered.txt
Thyagarajan2014/Thyagarajan2014-rep2_prefs_trimmed_renumbered.txt
Thyagarajan2014/Thyagarajan2014-rep3_prefs_trimmed_renumbered.txt
Thyagarajan2014/Thyagarajan2014-WSNHA_merged_prefs_renumbered.txt
```

Run `phydms` to test these models as experimental codon substitution models:

In [ ]:

```
#phydms_comprehensive for all ExpCM, no diffpref fitting:
run_directory = '%s/phylogenetics/' % base_directory
outprefix = 'phydms_output'
alignment = '%s/phylogenetics/H1_1prYearHostSubtype_alignment.fasta' % base_directory
allmodels = ' '.join(ExpCM_models)
avgrandcontrolfile = '%s/preferences/Overall-WSNHA_merged_prefs_renumbered.txt' % base_directory # this will be the only ExpCM that we test with randomized-across-sites or averaged-across-sites preferences
commands = ['%s %s %s --avgrandcontrol %s --no-diffprefsbysite' % (outprefix, alignment, allmodels, avgrandcontrolfile)]
RunScript(run_directory, 'phydmsNB', 'phydms_comprehensive', commands, True, 16, walltime=240)

#phydms to fit differential preferences for the overall merged model:
run_directory = '%s/phylogenetics/' % base_directory
alignment = '%s/phylogenetics/H1_1prYearHostSubtype_alignment.fasta' % base_directory
tree = '%s/phylogenetics/phydms_output_YNGKP_M0_tree.newick' % base_directory
expcm = 'ExpCM_%s/preferences/Overall-WSNHA_merged_prefs_renumbered.txt' % base_directory
outprefix = 'phydms_diffprefs_output_ExpCM_OverallMerge'
commands = ['%s %s %s %s --diffprefsbysite --seed 1' % (alignment, tree, expcm, outprefix)]
RunScript(run_directory, 'phyDiff', 'phydms', commands, True, 16, walltime=240)
```

Use `phydms_renumber` so that the results from `phydms` are numbered back to sequential numbering.

In [4]:

```
# make a renumbering file for phydms_renumber. This file lists on each line the old number, a space, and the new number (this goes in the opposite direction as the file used for dms_editsites):
f = open('%s/renumber_back_to_sequential.txt' % base_directory, 'w')
f.write("#renumbered_site sequential_site\n")
for original_number in range(2,566):
    f.write("%s %s\n" % (original_number-1, original_number))
f.close()

run_directory = '%s/phylogenetics/' % base_directory
renumberfile = '%s/renumber_back_to_sequential.txt' % base_directory

outputprefixes = ['%s/phylogenetics/phydms_output*bysite*.txt' % base_directory, '%s/phylogenetics/phydms_diffprefs_output*bysite*.txt' % base_directory]  

for outputprefix in outputprefixes:
    commands = ['%s %s' % (renumberfile, outputprefix)]
    RunScript(run_directory, 'phyrenumber', 'phydms_renumber', commands, False, 1)
```

```
Running phydms_renumber for phyrenumber in directory /fh/fast/bloom_j/grp/mike/2016_WSN_HA_analysis_forpaper/phylogenetics/...
Done running phydms_renumber for phyrenumber in directory /fh/fast/bloom_j/grp/mike/2016_WSN_HA_analysis_forpaper/phylogenetics/.

Running phydms_renumber for phyrenumber in directory /fh/fast/bloom_j/grp/mike/2016_WSN_HA_analysis_forpaper/phylogenetics/...
Done running phydms_renumber for phyrenumber in directory /fh/fast/bloom_j/grp/mike/2016_WSN_HA_analysis_forpaper/phylogenetics/.
```

Collect the model results; rescale the preferences from the best-performing model by the optimized stringency parameter and use for all subsequent analysis of preferences.

In [6]:

```
def ReadModelResults(modelprefix):
    '''Retrieves the log likelihood and optimized model parameters for the given model prefix *modelprefix*.
    Returns a dictionary keyed by `log likelihood`, `stringencyparameter`, etc, for all parameters provided in the
    *_modelparams file with the given model prefix. Also includes a key for `modelname`, which is extracted from the
    *modelprefix* assuming that it is preceded by 'phydms_output_'.
    
    *modelprefix* should provide the full path prefix to files ending with '_loglikelihood.txt' and '_modelparams.txt'.
    '''
    modelresults = {}
    with open('%s_modelparams.txt' % modelprefix) as infile:
            for line in infile:
                if line[0] != '#':
                    param, val = line.split(' = ')
                    modelresults[param] = float(val)
    with open('%s_loglikelihood.txt' % modelprefix) as infile:
            for line in infile:
                if line[0] != '#':
                    param, val = line.split(' = ')
                    modelresults[param] = float(val)
                    
    modelresults['modelname'] = modelprefix[modelprefix.find('phydms_output_')+14:].replace('trimmed','').replace('renumbered','').replace('prefs','').replace('_',' ').rstrip()
    modelresults['modelprefix'] = modelprefix
    return modelresults

# Collect model results for all models tested and format model names for presentation in a table
model_prefixes = glob.glob('%s/phylogenetics/phydms_output_*loglikelihood.txt' % base_directory)  #### this needs to be fixed for final version
model_prefixes = [ f.replace('_loglikelihood.txt', '') for f in model_prefixes]
model_result_list = []
for m in model_prefixes:
    model_result_list.append(ReadModelResults(m))

# sort models by log likelihood, best model first
model_result_list = sorted(model_result_list, key=lambda k: k['log likelihood'], reverse=True) 
print "Here are the models in order of performance:\n"
print "Model, log likelihood"
print "====================="
for m in model_result_list:
    print m['modelname'] + ', ' + str(m['log likelihood'])
print '\n'
print 'The best model (%s) used an optimized stringency parameter of %.2f.' % (model_result_list[0]['modelname'], model_result_list[0]['stringencyparameter'])
print 'Creating a rescaled preferences file using Overall-WSNHA_merged_prefs.txt, scaled by that stringency parameter.'
run_directory = 'preferences/'
input_file = '%s/preferences/Overall-WSNHA_merged_prefs.txt' % base_directory
rescaled_prefs_file = '%s/preferences/Overall-WSNHA_merged_prefs_rescaled.txt' % base_directory
beta = model_result_list[0]['stringencyparameter']
commands = ['%s rescale %s --stringencyparameter %s' % (rescaled_prefs_file, input_file, beta)]
RunScript(run_directory, 'rescaleprefs', 'dms_merge', commands, False, 1)
```

```
Here are the models in order of performance:

Model, log likelihood
=====================
ExpCM Overall-WSNHA merged, -14933.5
ExpCM 2016-WSNHA merged, -15032.3
ExpCM Thyagarajan2014-WSNHA merged, -15104.1
ExpCM 2016-WSNHA-rep1, -15163.6
ExpCM 2016-WSNHA-rep2, -15195.7
ExpCM Thyagarajan2014-rep3, -15249.7
ExpCM 2016-WSNHA-rep3, -15286.9
ExpCM Thyagarajan2014-rep1, -15348.4
ExpCM Thyagarajan2014-rep2, -15438.2
YNGKP M8, -16003.9
YNGKP M0, -16418.9
averaged ExpCM Overall-WSNHA merged, -16419.3
randomized ExpCM Overall-WSNHA merged, -16430.2


The best model (ExpCM Overall-WSNHA merged) used an optimized stringency parameter of 1.82.
Creating a rescaled preferences file using Overall-WSNHA_merged_prefs.txt, scaled by that stringency parameter.
Running dms_merge for rescaleprefs in directory preferences/...
Done running dms_merge for rescaleprefs in directory preferences/.
```

---

## Make figures¶

**Plot the fractions of each type of mutation in each of the samples:**

In [5]:

```
freqs_files = ['alignments/rep_%s_alignmentsummarymutfreqs.txt' % replicate for replicate in [1,2,3]]
data = {}
for f in freqs_files:
    with open(f) as infile:
        for line in infile:
            if line[0] == '#':
                headers = line[1:].split()[1:] # don't need the "sample" column header
            else:
                entries = line.split()
                sample_name = entries[0]
                data[sample_name] = {}
                for header, data_entry in zip(headers, entries[1:]):
                    data[sample_name][header] = float(data_entry)

#samples = sorted(data.keys())
samples = ['mutDNA1', 'mutvirus1', 'mutDNA2', 'mutvirus2', 'mutDNA3', 'mutvirus3', 'wtDNA', 'wtvirus']

synonymous = [data[sample]['synonymous'] for sample in samples]
nonsynonymous = [data[sample]['nonsynonymous'] for sample in samples]
stop = [data[sample]['stop_codon'] for sample in samples]
n1nts = [data[sample]['1_nucleotide_mutation'] for sample in samples]
n2nts = [data[sample]['2_nucleotide_mutations'] for sample in samples]
n3nts = [data[sample]['3_nucleotide_mutations'] for sample in samples]
ind = np.arange(len(samples))
spacing_width = 0.4
bar_width = 1*spacing_width
fig, ax = plt.subplots()
synbar = plt.bar(ind, synonymous, bar_width, color = orange, edgecolor='k')
nsbar = plt.bar(ind, nonsynonymous, bar_width, color = lightblue, bottom=synonymous, edgecolor='k')
stopbar = plt.bar(ind, stop, bar_width, color = 'red', bottom=[synonymous[i]+nonsynonymous[i] for i in range(len(samples))], edgecolor='k')
n1ntbar = plt.bar(ind+spacing_width, n1nts, bar_width, color = green, edgecolor='k')
n2ntbar = plt.bar(ind+spacing_width, n2nts, bar_width, color = yellow, bottom=n1nts, edgecolor='k')
n3ntbar = plt.bar(ind+spacing_width, n3nts, bar_width, color = purple, bottom=[n1nts[i]+n2nts[i] for i in range(len(samples))], edgecolor='k')
spineOffset = {'left': 5, 'bottom': 5}    
[spine.set_position(('outward',spineOffset[loc])) if loc in ['left','bottom'] else spine.set_color('none') for loc, spine in ax.spines.items() ] 
plt.rcParams['xtick.direction'] = 'out'
plt.rcParams['ytick.direction'] = 'out'
ax.xaxis.set(ticks_position = 'bottom')
ax.yaxis.set(ticks_position = 'left')
ax.set_xticks(ind + spacing_width)
#ax.set_xticklabels(samples)
ax.set_xticklabels(['mutant\nplasmid 1', 'mutant\nvirus 1', 'mutant\nplasmid 2', 'mutant\nvirus 2', 'mutant\nplasmid 3', 'mutant\nvirus 3', 'wt\nplasmid', 'wt\nvirus'])
yformatter = plt.ScalarFormatter(useMathText=True)
yformatter.set_powerlimits((-2, 2))
ax.yaxis.set_major_formatter(yformatter)
yticker = MaxNLocator(5)
ax.yaxis.set_major_locator(yticker)
ax.set_ylabel('per-codon mutation frequency', fontsize=18)
labels = ax.get_xticklabels()
#plt.setp(labels, rotation=90, fontsize=18)
plt.setp(labels, rotation=90, fontsize=16)
labels = ax.get_yticklabels()
plt.setp(labels, fontsize=14)
ax.set_xlim([-0.05,8])
ax.legend((stopbar[0], nsbar[0], synbar[0], n3ntbar[0], n2ntbar[0], n1ntbar[0]), 
          ('stop', 'nonsyn.', 'syn.', '3 nt', '2 nt', '1 nt'),
         fontsize=13)
fig.set_size_inches(6,5)
plt.tight_layout()
filepath = 'notebook_figures/mutationfrequency_by_sample.pdf'
plt.savefig(filepath)
png = filepath.rstrip('.pdf') + '.png'
!convert -density 192 -trim $filepath $png
display(Image(png, width=500))
```

**Plot stop codon frequencies in each mutant virus library relative to the respective plasmid mutant library:**

In [28]:

```
def GetTotalMuttypeFreq(counts_file, muttype):
    # Returns the overall frequency of a particular type of
    # mutation (*muttype*) in a codon counts file.
    # *muttype* should be 'STOP', 'NS', or 'SYN'
    counts = dms_tools.utils.ClassifyCodonCounts(
        dms_tools.file_io.ReadDMSCounts(counts_file, 'codon'))
    return float(counts['TOTAL_%s' % muttype])/counts['TOTAL_COUNTS']

def CalcMuttypePurgeFreq(mutvirus, wtvirus, mutDNA, wtDNA, muttype):
    # Returns the fraction of *muttype* mutations that are purged during selection,
    # defined by:
    # 1 - (f_mutvirus - f_wtvirus)/(f_mutDNA - f_wtDNA)
    # where *f_sample* is the overall frequency of *muttype* codons in *sample*,
    # and the wtvirus and wtDNA samples are used to correct for the erroneous
    # rate of observing *muttype* mutations due to PCR and sequencing errors in 
    # virus and DNA samples, respectively.
    # First four calling arguments are paths to codoncounts files.
    # *muttype* should be 'STOP', 'NS', or 'SYN'
    numerator = GetTotalMuttypeFreq(mutvirus, muttype) - GetTotalMuttypeFreq(wtvirus, muttype)
    denominator = GetTotalMuttypeFreq(mutDNA, muttype) - GetTotalMuttypeFreq(wtDNA, muttype)
    return 1 - numerator/denominator

# specify codon counts files
new2016_wtDNA_file = 'alignments/wtDNAcounts.txt'
new2016_wtvirus_file = 'alignments/wtviruscounts.txt'
new2016_mutDNA_files = ['alignments/mutDNA%scounts.txt' % r for r in [1,2,3]]
new2016_mutvirus_files = ['alignments/mutvirus%scounts.txt' % r for r in [1,2,3]]
old2014_wtDNA_files = ['Thyagarajan2014/replicate_%s_DNA_codoncounts.txt' % r for r in [1,2,3]]
old2014_wtvirus_files = ['Thyagarajan2014/replicate_%s_virus_codoncounts.txt' % r for r in [1,2,3]]
old2014_mutDNA_files = ['Thyagarajan2014/replicate_%s_mutDNA_codoncounts.txt' % r for r in [1,2,3]]
old2014_mutvirus_files = ['Thyagarajan2014/replicate_%s_mutvirus_codoncounts.txt' % r for r in [1,2,3]]

old_replicates_stop_purges = [1-CalcMuttypePurgeFreq(old2014_mutvirus_files[i], old2014_wtvirus_files[i], old2014_mutDNA_files[i], old2014_wtDNA_files[i], 'STOP') for i in [0,1,2]]
new_replicates_stop_purges = [1-CalcMuttypePurgeFreq(new2016_mutvirus_files[i], new2016_wtvirus_file, new2016_mutDNA_files[i], new2016_wtDNA_file, 'STOP') for i in [0,1,2]]

f, ax = plt.subplots()
index = [1, 1, 1, 1.2, 1.2, 1.2]
opacity = 1
two_group_colors = ['red','blue']
six_group_colors = [two_group_colors[0] for i in range(3)] + [two_group_colors[1] for k in range(3)]
ax.scatter(index, old_replicates_stop_purges + new_replicates_stop_purges, marker='o', alpha=opacity, 
                    color=six_group_colors, s=70,
                    edgecolor='black', linewidth=1.0)
means = [np.mean(old_replicates_stop_purges), np.mean(new_replicates_stop_purges)]
ax.scatter([1,1.2], means, marker='_', color='k',
          s=270, linewidth=2.5)
ax.set_xticks([1, 1.2])
ax.set_xticklabels(['old','new'])
spineOffset = {'left': 5, 'bottom': 5}    
[spine.set_position(('outward',spineOffset[loc])) if loc in ['left','bottom'] else spine.set_color('none') for loc, spine in ax.spines.items() ]         
ax.set_xlim( left=0.95, right=1.25 )
ax.set_ylim( bottom=0, top = 1.2*np.max(old_replicates_stop_purges + new_replicates_stop_purges))
ax.xaxis.set(ticks_position = 'bottom')
ax.yaxis.set(ticks_position = 'left', major_locator = MaxNLocator(4, prune=None))
plt.setp(ax.get_xticklabels(), fontsize=14, rotation=90)
(t, p) = scipy.stats.ttest_ind(old_replicates_stop_purges, new_replicates_stop_purges)
ax.text(0,.9,r"$p=%.3f$" % p, fontsize=11, transform=ax.transAxes)
plt.rcParams['xtick.direction'] = 'out'
plt.rcParams['ytick.direction'] = 'out'
ax.set_ylabel('relative stop codon\nfrequency after selection', fontsize=14)
f.set_size_inches([1.8,3.6])
plt.tight_layout()
filepath = 'notebook_figures/purge_stop_codons.pdf'
plt.savefig(filepath)
png = filepath.rstrip('.pdf') + '.png'
!convert -density 192 -trim $filepath $png
display(Image(png, width=200))
```

**Plot normalized synonymous mutation frequencies at each site for the old and new replicates:**

In [43]:

```
def ParseMutationFreqs(counts_file, parse_sites, muttype, err_correction_file=None, norm_to_sample=False):
    # pass *muttype* "NS" or "SYN" or "STOP"
    # if *err_correction_file* is specified, the frequency of muttype in that file (eg. the wildtype virus sequencing control)
    # is subtracted from the frequency in *counts_file* (eg. the mutant virus library) to account for
    # systematic errors arising at any point between RNA isolation and sequencing.
    
    # If norm_to_sample is set to True, the returned mutation frequencies at each site will be normalized by dividing
    # by the sum of those frequencies across all sites, making the area under the curve equal to one.
    counts = dms_tools.utils.ClassifyCodonCounts(
        dms_tools.file_io.ReadDMSCounts(counts_file, 'codon'))
    if err_correction_file:
        err_correction_counts = dms_tools.utils.ClassifyCodonCounts(
        dms_tools.file_io.ReadDMSCounts(err_correction_file, 'codon'))
    parse_sites = [str(site) for site in parse_sites]
    for site in parse_sites:
        assert site in counts
        if err_correction_file:
            assert site in err_correction_counts 
    freqs = []
    for site in parse_sites:
        # calculate the frequency of *muttype* mutation at each site, and optionally correct with control frequency
        if err_correction_file:
            err_correction_freq = float(err_correction_counts[site]['N_%s' % muttype])/err_correction_counts[site]['COUNTS']
        else:
            err_correction_freq = 0.0
        freqs.append( float(counts[site]['N_%s' % muttype])/counts[site]['COUNTS'] - err_correction_freq )                    
    if norm_to_sample:
        overall_mut_rate = sum(freqs) / float( len(freqs) ) 
    else:
        overall_mut_rate = 1
    freqs = [float(freq)/overall_mut_rate for freq in freqs]
    return freqs

# dictionary specifying number of possible synonymous mutation for each codon:
number_syn_possible = {}
for c in dms_tools.codons:
    number_syn_possible[c] = len(dms_tools.aa_to_codons[dms_tools.codon_to_aa[c]]) - 1

def UniformSynonymousSampling(counts_file, parse_sites, norm_to_sample=False):
    '''Simulates synonymous sampling rates for uniform sampling of all codons. 
    Only uses *counts_file* to get the wt codons at each site in order to determine 
    the number of possible synonymous mutations for each site.'''
    counts = dms_tools.file_io.ReadDMSCounts(counts_file, 'codon')
    parse_sites = [str(site) for site in parse_sites]
    for site in parse_sites:
            assert site in counts
    samplings = []
    for site in parse_sites:
        samplings.append(number_syn_possible[counts[site]['WT']])
    if norm_to_sample:
        overall_mut_rate = sum(samplings) / float( len(samplings) ) 
    else:
        overall_mut_rate = 1
    samplings = [float(sample_i)/overall_mut_rate for sample_i in samplings]
    return samplings

# Plot for mutant virus libraries:
sites = range(2,566)
old2014_norm_syn_freqs = [ParseMutationFreqs('Thyagarajan2014/replicate_%s_mutvirus_codoncounts.txt' % i, sites, 'SYN', 
                                             'Thyagarajan2014/replicate_%s_virus_codoncounts.txt' % i, norm_to_sample=True) for i in [1,2,3]]
new2016_norm_syn_freqs = [ParseMutationFreqs('alignments/mutvirus%scounts.txt' % i, sites, 'SYN', 
                                             'alignments/wtviruscounts.txt', norm_to_sample=True) for i in [1,2,3]]
uniform_simulated_norm_syn_freqs = UniformSynonymousSampling('alignments/wtviruscounts.txt', 
                                              sites, norm_to_sample=True)
all_datasets = [uniform_simulated_norm_syn_freqs] + old2014_norm_syn_freqs + new2016_norm_syn_freqs
labels = ['uniform', 'old replicate 1', 'old replicate 2', 'old replicate 3', 
          'new replicate 1', 'new replicate 2', 'new replicate 3']

colors = ['black'] + ['red' for i in range(3)] + ['blue' for i in range(3)] 

fig, axes = plt.subplots(7)
for i, data_i in enumerate(all_datasets):
    for j, background_data_j in enumerate(all_datasets):
        if i != j:
            axes[i].plot(sites, background_data_j, linewidth=0.9, color=colors[j], alpha=0.3)
    axes[i].plot(sites, data_i, linewidth=1.2, color=colors[i], alpha=1)
    spineOffset = {'left': 5, 'bottom': 1}
    [spine.set_position(('outward',spineOffset[loc])) if loc in ['left','bottom'] else spine.set_color('none') for loc, spine in axes[i].spines.items()]         
    axes[i].xaxis.set( ticks_position = 'bottom', major_locator = MaxNLocator(6, prune=None))
    axes[i].yaxis.set( ticks_position = 'left', major_locator = MaxNLocator(2, prune=None))
    axes[i].set_xlim(left=1, right=566)
    axes[i].set_ylim(bottom=-0.5, top=18)
    axes[i].text(0.2,0.60,labels[i], fontsize=14, transform=axes[i].transAxes, color=colors[i])
    if i<6:
        axes[i].set_xticklabels([])
        axes[i].set_yticklabels([])
        axes[i].spines['left'].set_visible(False)
        axes[i].yaxis.set_major_locator(plt.NullLocator())
    else:
        axes[i].set_yticklabels([])
        axes[i].spines['left'].set_visible(False)
        axes[i].yaxis.set_major_locator(plt.NullLocator())

plt.xlabel("codon site", fontsize = 18)
fig.text(0.09, 0.5, 'normalized synonymous frequency\nin mutant virus libraries', ha='center', va='center', rotation='vertical', fontsize=18)
plt.rcParams['xtick.direction'] = 'out'
plt.rcParams['ytick.direction'] = 'out'
fig.set_size_inches([8,6])
filepath = 'notebook_figures/norm_synonymous_peaks.pdf'
plt.savefig(filepath)
png = filepath.rstrip('.pdf') + '.png'
!convert -density 192 -trim $filepath $png
display(Image(png, width=800))

# Standard deviations of per-site normalized synonymous mutation frequencies:
print "Standard deviations of normalized synonymous frequencies:"
print "old replicates:"
print np.std(old2014_norm_syn_freqs[0]), np.std(old2014_norm_syn_freqs[1]), np.std(old2014_norm_syn_freqs[2])
print "old replicates, as mean +/- sd:"
print np.mean([np.std(old2014_norm_syn_freqs[0]), np.std(old2014_norm_syn_freqs[1]), np.std(old2014_norm_syn_freqs[2])]), "+/-", np.std([np.std(old2014_norm_syn_freqs[0]), np.std(old2014_norm_syn_freqs[1]), np.std(old2014_norm_syn_freqs[2])])
print "new replicates:"
print np.std(new2016_norm_syn_freqs[0]), np.std(new2016_norm_syn_freqs[1]), np.std(new2016_norm_syn_freqs[2])
print "new replicates, as mean +/- sd:"
print np.mean([np.std(new2016_norm_syn_freqs[0]), np.std(new2016_norm_syn_freqs[1]), np.std(new2016_norm_syn_freqs[2])]), "+/-", np.std([np.std(new2016_norm_syn_freqs[0]), np.std(new2016_norm_syn_freqs[1]), np.std(new2016_norm_syn_freqs[2])])
```

```
Standard deviations of normalized synonymous frequencies:
old replicates:
1.49066007433 1.56579518362 1.82838916992
old replicates, as mean +/- sd:
1.62828147596 +/- 0.144784064618
new replicates:
1.14164882824 1.14034539049 1.25909347057
new replicates, as mean +/- sd:
1.1803625631 +/- 0.0556737016366
```

In [8]:

```
# Plot for mutant DNA libraries, to make sure the bottleneck peaks are not the result of unbalanced mutation frequencies in the starting mutant DNA libraries:
sites = range(2,566)
old2014_norm_syn_freqs = [ParseMutationFreqs('Thyagarajan2014/replicate_%s_mutDNA_codoncounts.txt' % i, sites, 'SYN', 
                                             'Thyagarajan2014/replicate_%s_DNA_codoncounts.txt' % i, norm_to_sample=True) for i in [1,2,3]]
new2016_norm_syn_freqs = [ParseMutationFreqs('alignments/mutDNA%scounts.txt' % i, sites, 'SYN', 
                                             'alignments/wtDNAcounts.txt', norm_to_sample=True) for i in [1,2,3]]
uniform_simulated_norm_syn_freqs = UniformSynonymousSampling('alignments/wtDNAcounts.txt', 
                                              sites, norm_to_sample=True)
all_datasets = [uniform_simulated_norm_syn_freqs] + old2014_norm_syn_freqs + new2016_norm_syn_freqs
labels = ['uniform', 'old replicate 1', 'old replicate 2', 'old replicate 3', 
          'new replicate 1', 'new replicate 2', 'new replicate 3']

colors = ['black'] + ['firebrick' for i in range(3)] + ['dodgerblue' for i in range(3)] 

fig, axes = plt.subplots(7)
for i, data_i in enumerate(all_datasets):
    for j, background_data_j in enumerate(all_datasets):
        if i != j:
            axes[i].plot(sites, background_data_j, linewidth=0.9, color=colors[j], alpha=0.3)
    axes[i].plot(sites, data_i, linewidth=1.2, color=colors[i], alpha=1)
    spineOffset = {'left': 5, 'bottom': 1}
    [spine.set_position(('outward',spineOffset[loc])) if loc in ['left','bottom'] else spine.set_color('none') for loc, spine in axes[i].spines.items()]         
    axes[i].xaxis.set( ticks_position = 'bottom', major_locator = MaxNLocator(6, prune=None))
    axes[i].yaxis.set( ticks_position = 'left', major_locator = MaxNLocator(2, prune=None))
    axes[i].set_xlim(left=1, right=566)
    axes[i].set_ylim(bottom=-0.5, top=18)
    axes[i].text(0.2,0.60,labels[i], fontsize=14, transform=axes[i].transAxes, color=colors[i])
    if i<6:
        axes[i].set_xticklabels([])
        axes[i].set_yticklabels([])
        axes[i].spines['left'].set_visible(False)
        axes[i].yaxis.set_major_locator(plt.NullLocator())
    else:
        axes[i].set_yticklabels([])
        axes[i].spines['left'].set_visible(False)
        axes[i].yaxis.set_major_locator(plt.NullLocator())

plt.xlabel("codon site", fontsize = 18)
fig.text(0.09, 0.5, 'normalized synonymous frequency\nin mutant plasmid libraries', ha='center', va='center', rotation='vertical', fontsize=18)
plt.rcParams['xtick.direction'] = 'out'
plt.rcParams['ytick.direction'] = 'out'
fig.set_size_inches([8,6])
filepath = 'notebook_figures/norm_synonymous_peaks_mutDNA.pdf'
plt.savefig(filepath)
png = filepath.rstrip('.pdf') + '.png'
!convert -density 192 -trim $filepath $png
display(Image(png, width=800))
```

**Plot correlations in amino-acid preferences between replicates for the old and new experiments:**

In [25]:

```
def PlotPreferencesCorrelation(preffile1, preffile2, label_1, label_2, plotsites, outfile, c='k'):
    # plot correlation between the preferences for all amino acids at the
    # sites listed in *plotsites*.    
    (sites_file1, wts_file1, pi_means_file1, pi_95credint_file1, h_file1) = dms_tools.file_io.ReadPreferences(preffile1)
    (sites_file2, wts_file2, pi_means_file2, pi_95credint_file2, h_file2) = dms_tools.file_io.ReadPreferences(preffile2)
    plotsites = [str(site) for site in plotsites]
    for site in plotsites:
        assert site in sites_file1
        assert site in sites_file2
    xs = []
    ys = []
    for site in plotsites:
        for aa in dms_tools.aminoacids_nostop:
            xs.append(pi_means_file1[site][aa])
            ys.append(pi_means_file2[site][aa])
    
    (r, p) = scipy.stats.pearsonr(xs, ys)
    
    fig = plt.figure()
    plt.plot(xs, ys, 
             marker='o', linestyle='None', color=c, 
             markeredgewidth=0.0, alpha = 0.35, markersize=6)
    plt.gca().set_ylim(bottom=-0.01, top=1)
    plt.gca().set_xlim(left=-0.01, right=1)
    spineOffset = {'left': 5, 'bottom': 5}    
    [spine.set_position(('outward',spineOffset[loc])) if loc in ['left','bottom'] else spine.set_color('none') for loc, spine in plt.gca().spines.items() ]         
    plt.rcParams['xtick.direction'] = 'out'
    plt.rcParams['ytick.direction'] = 'out'
    plt.gca().xaxis.set( ticks_position = 'bottom', major_locator = MaxNLocator(3, prune=None))
    plt.gca().yaxis.set( ticks_position = 'left', major_locator = MaxNLocator(3, prune=None)) 
    plt.xlabel(label_1, fontsize=28)
    plt.ylabel(label_2, fontsize=28)
    plt.setp(plt.gca().get_xticklabels(), fontsize=22)
    plt.setp(plt.gca().get_yticklabels(), fontsize=22)
    rstring = r'$R^2\!=%.2f$' % (r**2)
    if p < 1e-10:
        pstring = '$p < 10^{-10}$'
    else:
        pstring = '$p = %s$' % dms_tools.plot.Base10Formatter(p, 2, 1, 2)    
    annotation = '%s\n%s' % (rstring, pstring)
    plt.gca().text(0.18,0.8, annotation,size=24,)
    fig.set_size_inches([4.5,4.5])
    plt.tight_layout()
    filepath = 'notebook_figures/%s.pdf' % outfile
    plt.savefig(filepath)
    png = filepath.rstrip('.pdf') + '.png'
    !convert -density 192 -trim $filepath $png
    display(Image(png, width=250))

old_method_replicates = ['Thyagarajan2014/Thyagarajan2014-rep%s_prefs.txt' % i for i in [1,2,3]]
new_method_replicates = ['preferences/2016-WSNHA-rep%s_prefs.txt' % i for i in [1,2,3]]

for i in range(len(old_method_replicates)):
    for j in range(len(old_method_replicates)):
        if i < j:
            PlotPreferencesCorrelation(old_method_replicates[i],  
                                       old_method_replicates[j], 
                                       'old replicate %s' % (i+1), 
                                       'old replicate %s' % (j+1), 
                                       range(2,566), 'old_corr_rep_%s_vs_%s' % (i+1, j+1),
                                       c = 'red')

for i in range(len(new_method_replicates)):
    for j in range(len(new_method_replicates)):
        if i < j:
            PlotPreferencesCorrelation(new_method_replicates[i],  
                                       new_method_replicates[j], 
                                       'new replicate %s' % (i+1), 
                                       'new replicate %s' % (j+1), 
                                       range(2,566), 'new_corr_rep_%s_vs_%s' % (i+1, j+1),
                                       c='blue')
```

**Model site entropy of amino-acid preferences as a function of relative solvent accessability and belonging to antigenic sites or particular domains of HA:**
PDB file `1RVX` was manually renumbered to sequential numbering to make the file `PDB_structure/1RVX_trimer_sequentialnumbering.pdb`, and the DSSP webserver (http://swift.cmbi.ru.nl/gv/dssp/) was used with this file to make `PDB_structure/1RVX_trimer_sequentialnumbering.dssp`.

In [4]:

```
# This section has a lot of output from the statistical models; saving to a log file.
log = open('%s/notebook_figures/entropy_rsa_stats.log' % base_directory, 'w')

# A function to plot RSA vs. site entropy, highlight selected sites, and add an annotation with the results from multiple linear regression
def Scatterplot(entropies_list, rsas_list, selecteds_list, title, outfile, annotation=None, select_color='darkorange'):
    fig = plt.figure()
    gs = gridspec.GridSpec(2, 1, height_ratios=[6, 1.5])
    plotax = plt.subplot(gs[0]) # for the plot
    textax = plt.subplot(gs[1]) # for a text annotation of the results of the model
    unselected_xs = []
    unselected_ys = []
    selected_xs = []
    selected_ys = []
    for i, (rsa, entropy) in enumerate(zip(rsas_list,entropies_list)):
        if selecteds_list[i] == 0:
            unselected_xs.append(rsa)
            unselected_ys.append(entropy)
        elif selecteds_list[i] == 1:
            selected_xs.append(rsa)
            selected_ys.append(entropy)
        else:
            raise ValueError("selecteds_list should only contain 0 or 1 at each site.")
    
    plotax.plot(unselected_xs, unselected_ys, 
             marker='o', linestyle='None', color='black', 
             markeredgewidth=0.0, alpha = 0.3, markersize=5)
    plotax.plot(selected_xs, selected_ys,
             marker='o', linestyle='None', color=select_color, 
             markeredgewidth=0.5, alpha = 1, markersize=6)
    
    plotax.set_ylim( bottom=0, top=4.4)
    plotax.set_xlim( left=-0.02, right=1 )
    
    # clean up chart junk
    spineOffset = {'left': 5, 'bottom': 0}    
    [spine.set_position(('outward',spineOffset[loc])) if loc in ['left','bottom'] else spine.set_color('none') for loc, spine in plotax.spines.items() ]         
    for loc, spine in textax.spines.items():
        spine.set_color('none')
    for i in textax.get_xticklabels() + textax.get_yticklabels() + textax.get_xticklines() + textax.get_yticklines():
        i.set_visible(False)
    plt.rcParams['xtick.direction'] = 'out'
    plt.rcParams['ytick.direction'] = 'out'
    plotax.xaxis.set( ticks_position = 'bottom', major_locator = MaxNLocator(5, prune=None))
    plotax.yaxis.set( ticks_position = 'left', major_locator = MaxNLocator(5, prune=None)) 
    plotax.set_xlabel("RSA", fontsize=18)
    plotax.set_ylabel("site entropy", fontsize=18)
    
    fig.set_size_inches([4,4])
    
    if annotation:
        textax.text(-0.1,0,annotation,fontsize=12)
    
    plt.tight_layout()
    filepath = 'notebook_figures/%s.pdf' % outfile
    plt.savefig(filepath)
    png = filepath.rstrip('.pdf') + '.png'
    !convert -density 192 -trim $filepath $png
    display(Image(png, width=400))

# Define groups of sites of interest based on structural, functional, and antigenic properties:
def GetSites(sites_file):
    lines = [line for line in open(sites_file).readlines() if (not line.isspace()) and line[0] != '#']
    selectedsites = [str(int(line.split()[0])) for line in lines]
    return selectedsites
antigenic_sites = GetSites('Thyagarajan2014/Caton_H1_HA_antigenic_sites.txt')
nearby_antigenic_sites = GetSites('Thyagarajan2014/nearby_antigenic_sites.txt')
all_rbs_sites = GetSites('Thyagarajan2014/allRBS_residues.txt')
conserved_rbs_sites = GetSites('Thyagarajan2014/receptor_binding_residues.txt')
# H3 and H1 antigenic cluster jump sites (in sequential numbering) defined by (Koel et al, 2013):
koel_et_al_sites = ['202','206','169','172','171','168','158']
# union of sites in the mostly overlapping epitopes of broadly-neutralizing stem-binding antibodies FI6v3, F10, CR6261, and CR9114:
stem_epitope_union = ['332', '392', '391', '396', '395', '399', '25', '48', '49', '46', '47', '45', '400', '364', '362', 
                      '363', '361', '305', '306', '307', '381', '382', '384', '385', '386', '388', '389', '379']
# subdomain boundaries (in sequential numbering) defined in (Gamblin et al, 2004):
HA1_fusionDomain_sites = range(18,72+1) + range(291, 340+1)
HA1_fusionDomain_sites = [str(r) for r in HA1_fusionDomain_sites]
HA1_VestigialEsteraseDomain_sites = range(73,125+1) + range(279,290+1)
HA1_VestigialEsteraseDomain_sites = [str(r) for r in HA1_VestigialEsteraseDomain_sites]
HA1_ReceptorBindingDomain_sites = range(126, 278+1)
HA1_ReceptorBindingDomain_sites = [str(r) for r in HA1_ReceptorBindingDomain_sites]
HA2_fusionDomain_sites = range(344, 503+1)
HA2_fusionDomain_sites = [str(r) for r in HA2_fusionDomain_sites]
selected_site_groups = [("Caton et al. antigenic sites", antigenic_sites), 
                        ("antigenic sites plus contacting residues", nearby_antigenic_sites), 
                        ("conserved receptor binding sites", conserved_rbs_sites), 
                        ("all receptor binding sites", all_rbs_sites),
                        ("antigenic cluster jump sites", koel_et_al_sites), 
                        ("stalk epitopes", stem_epitope_union),
                        ("HA1 fusion domain", HA1_fusionDomain_sites), 
                        ("HA1 vestigial esterase domain", HA1_VestigialEsteraseDomain_sites),
                        ("HA1 receptor binding domain", HA1_ReceptorBindingDomain_sites),
                        ("HA2 fusion domain", HA2_fusionDomain_sites)]


# sites to include in statistical tests (all sites)
testsites = [str(i) for i in range(2,566)]

# preferences file to use for site entropy information
prefs_file = 'preferences/Overall-WSNHA_merged_prefs_rescaled.txt'
log.write('Obtaining site entropy information from preferences file %s.\n' % prefs_file)
entropies = dms_tools.file_io.ReadPreferences(prefs_file)[4]

# Parse relative solvent accessability for sites present in the 1RVX crystal structure:
dssp = mapmuts.dssp.ReadDSSP('PDB_structure/1RVX_trimer_sequentialnumbering.dssp','Tien2013','A')
dssp_sites = [str(i) for i in sorted(dssp.keys())]

sharedsites = [r for r in testsites if (r in entropies) and (r in dssp_sites)] # sites with information for both entropy and RSA
log.write('%d sites have both entropy and RSA information and thus will be included in the model.\n' % len(sharedsites))
# lists of data for sites in sharedsites
entropies_list = [entropies[r] for r in sharedsites]
rsas_list = [dssp[int(r)]['RSA'] for r in sharedsites]

# do multiple linear regression for each of the site groups:
for (sites_label, selectedsites) in selected_site_groups:
    log.write("Testing selected sites in group %s.\n" % sites_label)
    selectedsites = [r for r in selectedsites if r in sharedsites] # can only consider selectedsites that have both entropy and RSA values
    selecteds_list = [int(r in selectedsites) for r in sharedsites] # 0 or 1 for each site in sharedsites designating if the site is in the selected group

    # make dataframe containing entropy, RSA, and a binary classification 'selected' (0 or 1) for all sites in sharedsites
    d = {'entropy' : entropies_list,
         'rsa' : rsas_list,
         'selected' : selecteds_list}
    df = pd.DataFrame(d)

    model = smf.ols(formula='entropy ~ rsa + selected', data=df)
    result = model.fit()

    log.write("%s"%result.summary())
    
    selected_coef = result.params['selected']
    selected_sterr = result.bse['selected']
    selected_p = result.pvalues['selected']
    rsa_coef = result.params['rsa']
    rsa_sterr = result.bse['rsa']
    rsa_p = result.pvalues['rsa']

    annotation_string = "RSA:\n  coef=%.2g, stderr=%.2g, p=%.2g\n%s:\n  coef=%.2g, stderr=%.2g, p=%.2g" % (
    rsa_coef, rsa_sterr, rsa_p, sites_label, selected_coef, selected_sterr, selected_p) 

    plot_title = sites_label

    select_site_color = 'orangered'
    if sites_label == "HA1 fusion domain":
        select_site_color = 'magenta'
    if sites_label == 'HA1 vestigial esterase domain':
        select_site_color = 'orange'
    if sites_label == 'HA1 receptor binding domain':
        select_site_color = 'dodgerblue'
    if sites_label == 'HA2 fusion domain':
        select_site_color = 'red'

    filelabel = sites_label.replace(' ','').replace('.', '')
    filelabel = 'RSA_entropy_%s' % filelabel
    Scatterplot(entropies_list,rsas_list,selecteds_list, plot_title, filelabel, annotation_string, select_color = select_site_color)

log.close()
```

Make logoplots of the rescaled preferences for the best model (Overall merged preferences), using both sequential and H3 numbering. The conversion between sequential H1 and conventional H3 numbering is given in `H1toH3_editsites.txt`.

An extra track above the preferences labeled "SEQ" is added to manually overlay the wild-type sequence. For now, it is generated using the secondary structure information.

In [34]:

```
# mapping from H1 sequential to H3 numbering:
files_to_renumber = ['%s/preferences/Overall-WSNHA_merged_prefs.txt' % base_directory, '%s/preferences/Overall-WSNHA_merged_prefs_rescaled.txt' % base_directory] 
run_directory = '%s/preferences/' % base_directory
for f_to_renumber in files_to_renumber:
    renumbered_file = f_to_renumber.replace(".txt", "_H3numbering.txt")
    commands = ['%s %s renumber %s/H1toH3_editsites.txt' % (f_to_renumber, renumbered_file, base_directory)]
    RunScript(run_directory, 'renumber', 'dms_editsites', commands, False, 1)

# Read the H1-to-H3 conversion in from H1toH3_editsites.txt:
H1_to_H3 = {} # keyed by string of H1 site, values are H3 site conversion.
with open('%s/H1toH3_editsites.txt' % base_directory) as infile:
        for line in infile:
            H1, H3 = line.split()
            H1_to_H3[H1] = H3
    
# Make RSA and SS files to annotate the logoplots with structural information.
RSA_outfile = open('%s/site_RSA.txt' % base_directory, 'w') 
RSA_H3_outfile = open('%s/site_RSA_H3numbering.txt' % base_directory, 'w') 
SS_outfile = open('%s/site_SS.txt' % base_directory, 'w')
SS_H3_outfile = open('%s/site_SS_H3numbering.txt' % base_directory, 'w')

dssp = mapmuts.dssp.ReadDSSP('PDB_structure/1RVX_trimer_sequentialnumbering.dssp','Tien2013','A')
for site in dssp.keys():
    site = str(site)
    site_H3number = H1_to_H3[site]
    RSA_outfile.write('%s %s\n' % (site, dssp[int(site)]['RSA']))
    RSA_H3_outfile.write('%s %s\n' % (site_H3number, dssp[int(site)]['RSA']))
    SS_outfile.write('%s %s\n' % (site, dssp[int(site)]['SS_CLASS']))
    SS_H3_outfile.write('%s %s\n' % (site_H3number, dssp[int(site)]['SS_CLASS']))

RSA_outfile.close()
RSA_H3_outfile.close()
SS_outfile.close()
SS_H3_outfile.close()

# dummy_file = '%s/placeholderfile.txt' % base_directory
# dummy_outfile = open(dummy_file, 'w')
# dummy_outfile.write('18 0\n')
# dummy_outfile.close()


#Make logoplots:
files_to_logoplot = files_to_renumber + [f.replace(".txt", "_H3numbering.txt") for f in files_to_renumber]
for f in files_to_logoplot:
    logoplot = f.replace('.txt','logo.pdf')
    if "H3numbering" in f:
        RSA_file = '%s/site_RSA_H3numbering.txt' % base_directory
        SS_file = '%s/site_SS_H3numbering.txt' % base_directory
    else:
        RSA_file = '%s/site_RSA.txt' % base_directory
        SS_file = '%s/site_SS.txt' % base_directory
    commands = ['%s %s --nperline 63 --numberevery 5 --colormap brg --overlay2 %s RSA "relative solvent accessibility" --overlay3 %s SS "secondary structure"' % (f, logoplot, RSA_file, SS_file)]
    commands.append('--overlay1 %s SEQ "dummy"' % SS_file)
    RunScript(run_directory, 'logo', 'dms_logoplot', commands, False, 1)
    png = logoplot.rstrip('.pdf') + '.png'
    !convert -density 192 -trim $logoplot $png
    print "Here is %s:" % png[png.rfind('/')+1:]
    display(Image(png, width=1000))
```

```
Running dms_editsites for renumber in directory /fh/fast/bloom_j/grp/mike/2016_03_WSN_HA_analysis_forpaper/preferences/...
Done running dms_editsites for renumber in directory /fh/fast/bloom_j/grp/mike/2016_03_WSN_HA_analysis_forpaper/preferences/.

Running dms_editsites for renumber in directory /fh/fast/bloom_j/grp/mike/2016_03_WSN_HA_analysis_forpaper/preferences/...
Done running dms_editsites for renumber in directory /fh/fast/bloom_j/grp/mike/2016_03_WSN_HA_analysis_forpaper/preferences/.

Running dms_logoplot for logo in directory /fh/fast/bloom_j/grp/mike/2016_03_WSN_HA_analysis_forpaper/preferences/...
Done running dms_logoplot for logo in directory /fh/fast/bloom_j/grp/mike/2016_03_WSN_HA_analysis_forpaper/preferences/.

Here is Overall-WSNHA_merged_prefslogo.png:
```

```
Running dms_logoplot for logo in directory /fh/fast/bloom_j/grp/mike/2016_03_WSN_HA_analysis_forpaper/preferences/...
Done running dms_logoplot for logo in directory /fh/fast/bloom_j/grp/mike/2016_03_WSN_HA_analysis_forpaper/preferences/.

Here is Overall-WSNHA_merged_prefs_rescaledlogo.png:
```

```
Running dms_logoplot for logo in directory /fh/fast/bloom_j/grp/mike/2016_03_WSN_HA_analysis_forpaper/preferences/...
Done running dms_logoplot for logo in directory /fh/fast/bloom_j/grp/mike/2016_03_WSN_HA_analysis_forpaper/preferences/.

Here is Overall-WSNHA_merged_prefs_H3numberinglogo.png:
```

```
Running dms_logoplot for logo in directory /fh/fast/bloom_j/grp/mike/2016_03_WSN_HA_analysis_forpaper/preferences/...
Done running dms_logoplot for logo in directory /fh/fast/bloom_j/grp/mike/2016_03_WSN_HA_analysis_forpaper/preferences/.

Here is Overall-WSNHA_merged_prefs_rescaled_H3numberinglogo.png:
```

Make a logoplot of the differential preferences fitted to each sit (in H3 numbering) by phydms:

In [39]:

```
renumber_file = '%s/H1toH3_editsites.txt' % base_directory
run_directory = '%s/phylogenetics/' % base_directory

outputprefixes = ['%s/phylogenetics/renumbered_phydms_diffprefs_output*bysite*.txt' % base_directory]  

for outputprefix in outputprefixes:
    commands = ['%s %s --renumberedprefix H3numbering' % (renumber_file, outputprefix)]
    RunScript(run_directory, 'phyrenumber', 'phydms_renumber', commands, False, 1)
    
######

run_directory = '%s/phylogenetics' % base_directory
diffprefsbysitefile = '%s/phylogenetics/H3numbering_renumbered_phydms_diffprefs_output_ExpCM_OverallMerge_diffprefsbysite.txt' % base_directory
plotfile = '%s/phylogenetics/H3numberingdifferentialselection.pdf' % base_directory
commands = ['%s %s --nperline 63 --colormap brg --numberevery 5' % (diffprefsbysitefile, plotfile)]
RunScript(run_directory, 'difflogo', 'phydms_plotselection', commands, False, 1)

png = plotfile.rstrip('.pdf') + '.png'
!convert -density 192 -trim $plotfile $png
print "Here is %s:" % png[png.rfind('/')+1:]
display(Image(png, width=1000))
```

```
Running phydms_renumber for phyrenumber in directory /fh/fast/bloom_j/grp/mike/2016_03_WSN_HA_analysis_forpaper/phylogenetics/...
Done running phydms_renumber for phyrenumber in directory /fh/fast/bloom_j/grp/mike/2016_03_WSN_HA_analysis_forpaper/phylogenetics/.

Running phydms_plotselection for difflogo in directory /fh/fast/bloom_j/grp/mike/2016_03_WSN_HA_analysis_forpaper/phylogenetics...
Done running phydms_plotselection for difflogo in directory /fh/fast/bloom_j/grp/mike/2016_03_WSN_HA_analysis_forpaper/phylogenetics.

Here is H3numberingdifferentialselection.png:
```

In [ ]:
